# Measuring implementation in global mental health: validation of a pragmatic implementation science measure in eastern Ukraine using an experimental vignette design

**DOI:** 10.1186/s12913-019-4097-y

**Published:** 2019-04-29

**Authors:** E. E. Haroz, P. Bolton, A. J. Nguyen, C. Lee, S. Bogdanov, J. Bass, N. S. Singh, Benjamin Doty, L. Murray

**Affiliations:** 1 Department of Mental Health, Johns Hopkins Bloomberg School of Public Health, 624 N. Broadway 8th fl, Baltimore, MD 21205 USA; 2 Department of International Health, Johns Hopkins Bloomberg School of Public Health, Baltimore, USA; 3 University of Virginia Curry School of Education, Virginia, USA; 4 Center for Mental Health and Psychosocial Support National University of Kyiv-Mohyla, Kyiv-Mohyla, Ukraine

**Keywords:** Dissemination & Implementation, Measurement, Validation, Mixed-methods, Global mental health

## Abstract

**Background:**

There is mounting evidence supporting the effectiveness of task-shifted mental health interventions in low- and middle-income countries (LMIC). However, there has been limited systematic scale-up or sustainability of these programs, indicating a need to study implementation. One barrier to progress is a lack of locally relevant and valid implementation measures. We adapted an existing brief dissemination and implementation (D&I) measure which includes scales for acceptability, appropriateness, feasibility and accessibility for local use and studied its validity and reliability among a sample of consumers in Ukraine.

**Methods:**

Local qualitative data informed adaptation of the measure and development of vignettes to test the reliability and validity. Participants were veterans and internally displaced persons (IDPs) recruited as part of a separate validity study of adapted mental health instruments. We examined internal consistency reliability, test-retest reliability, and construct and criterion validity for each scale on the measure. We randomly assigned half the participants to respond to a vignette depicting existing local psychiatric services which we knew were not well regarded, while the other half was randomized to a vignette describing a potentially more well-implemented mental health service. Criterion validity was assessed by comparing scores on each scale by vignette and by overall summary ratings of the programs described in the vignettes.

**Results:**

*N =* 169 participated in the qualitative study and *N* = 153 participated in the validity study. Qualitative findings suggested the addition of several items to the measure and indicated the importance of addressing professionalism/competency of providers in both the scales and the vignettes. Internal consistency reliabilities ranged from *α* = 0.85 for *feasibility* to *α* = 0.91 for *appropriateness*. Test-rest reliabilities were acceptable to good for all scales (*rho:* 0.61–0.79). All scales demonstrated substantial and significant differences in average scores by vignette assignment (*ORs:* 2.21–5.6) and overall ratings (*ORs:* 5.1–14.47), supporting criterion validity.

**Conclusions:**

This study represents an innovative mixed-methods approach to testing an implementation science measure in contexts outside the United States. Results support the reliability and validity of most scales for consumers in Ukraine. Challenges included large amounts of missing data due to participants’ difficulties responding to questions about a hypothetical program.

**Electronic supplementary material:**

The online version of this article (10.1186/s12913-019-4097-y) contains supplementary material, which is available to authorized users.

## Background

In Western and other High Income Countries (HIC), measuring the key concepts of implementation science has been noted as challenging [1, 2]. Few measures have undergone rigorous testing. In a review of 104 measures of implementation domains, 49% of the measures reported information on reliability, 26% on structural validity, 18% on predictive validity and 4% on responsiveness [3]. Existing measures are usually long, focus only on one sub-domain per measure (i.e. organizational climate), and/or focus only on one specific type of respondent (i.e. providers or organizational level staff). Creating pragmatic implementation science measures that are both relevant and feasible is a major priority [4].

Adding to these challenges, almost all existing implementation measures were developed for use in HIC, and therefore are based on assumptions that do not hold in many Low and Middle-Income Countries (LMIC). For example, many measures assume an established health care system that includes mental health services as “standard care”. In most LMIC, mental health services are limited and may include diverse “counseling” offered usually through international or local non-governmental organizations (NGOs). Some sites may have urban inpatient hospitals or facilities or a limited number of beds within a general hospital for severe mental illness cases. Rarely, if ever, are services integrated into the general health system [5, 6]. Many existing implementation measures also refer to aspects of mental health care that are often irrelevant in these settings, such as utilization of continuing education opportunities, use of billing and reimbursement systems, or other opportunities and infrastructure that do not exist in LMIC. Key differences between high and low-income contexts are not currently accounted for in existing implementation measures. With the growing call for implementation research in global mental health [7, 8] it is critical to develop accurate measurement procedures and instruments in order to advance implementation science [3, 9–11].

The Applied Mental Health Research group (AMHR) at Johns Hopkins University has developed a measure to evaluate multiple implementation domains: *acceptability, appropriateness, feasibility* and *accessibility* [1] specific to LMIC [12, 13]. Our team created four versions of the measure for different stakeholder types (i.e., consumers, providers, organizations, policy). Briefly, these measures were developed using a logical framework approach, and populated with items based on several leading implementation science frameworks (CFIR; [14], RE-AIM [15], EPIS [16] as well as our own experience and consultations with experts. The various versions for different stakeholders were then piloted in Iraq and Myanmar (results unpublished), and revised. In this paper, we describe an adaptation and testing procedure to test the consumer level instrument in Ukraine. Our specific aims were to: 1) adapt the measure for each specific context; 2) develop vignettes depicting existing and hypothetical mental health care services to use as criteria in a testing study; and 3) test the reliability and validity of the measure among a sample of mental health service consumers in Ukraine using the vignettes. We hypothesized that scores on the scales on the Implementation measure being tested would be higher for the vignette-based description of a “well”-implemented mental health program compared to the vignette-based description of a “poorly”-implemented mental health program.

### Ethics

The study was approved by both the Johns Hopkins Bloomberg School of Public Health (JHU) and the National University of Kyiv-Mohyla (NaUKMA) Institutional Review Boards (IRBs). All participants provided informed verbal consent in order to ensure confidentiality as approved by the IRBs.

## Methods

We used a mixed-methods approach. Qualitative methods were used to inform adaptation of the pre-existing scales and the development of vignettes. Quantitative methods were used to test the reliability and validity of the measure in a larger sample of potential mental health service consumers in Ukraine.

### Study context

As a response to the *Maidan* violence and ongoing war, Ukrainian mental health professionals have sought training in treatment of trauma-related concerns. As part of a three-year project sponsored by the United States Agency for International Development Victims of Torture Fund (VOT) JHU has partnered with NaUKMA to train mental health providers and peer veteran counselors in the Common Elements Treatment Approach (CETA) and test the intervention in a randomized controlled trial. As part of this study, local partners expressed great interest in learning more about the implementation of such programs to inform future scale-up and sustainability.

**Fig. 1:**
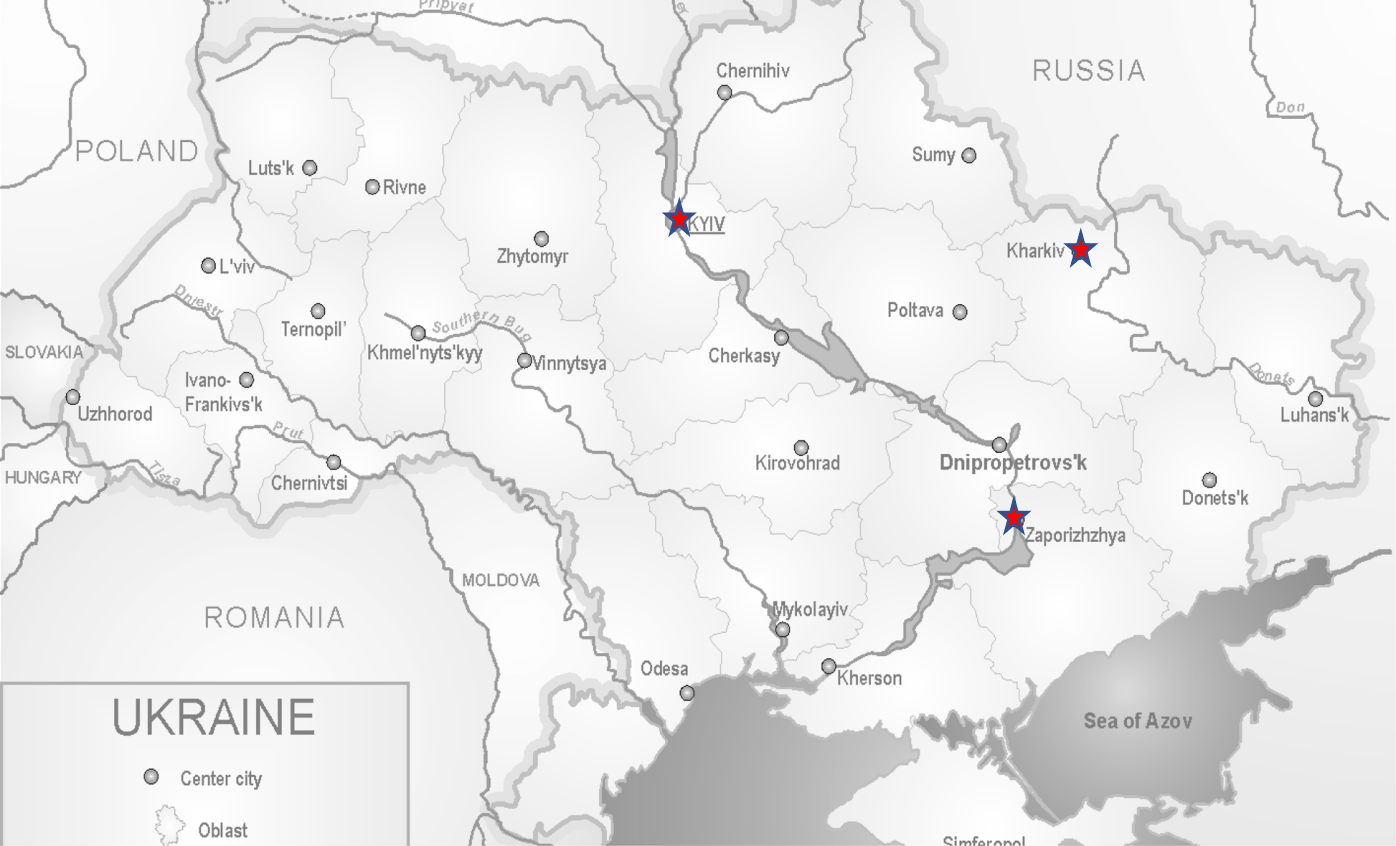
Map of study sites [38]

This study took place in three sites: Kharkiv (Aim 1); Kyiv (Aim 2); and Zaporizhia (Aims 1 & 2). The oblasts of Kharkiv and Zaporizhia are both adjacent to the Donbass region, the location of the ongoing conflict. All three cities have large numbers of Internally Displaced Persons (IDPs) and Veterans – the target population of the overall VOT-funded project (Fig. 1).

### Qualitative study to adapt implementation measures and develop validation vignettes

#### Research design

This study was built on two separate methodologies: a) a vignette based validation process used in the US for the validation of implementation measures [17]; and b) rapid ethnographic methods that we have used to develop and test mental health assessments in LMIC [18]. As part of the standard DIME (Design Implementation Monitoring and Evaluation) process we used qualitative methods – Free Listing (FL), and Focus Groups Discussions (FGDs) – to better understand local mental health and psychosocial problems and their solutions. For the current study, we added questions to both the FL and FGD guide (detailed below and in Additional file 1 and Additional file 2) to inform adaptations to the implementation measure and development of vignettes. We selected these approaches based on our experience doing mental health work in LMIC and feedback from local partners, suggesting that the limited mental health services available in most LMIC may result in consumers who are unfamiliar with a range in types, and qualities, of mental health services.

### Free listing and focus groups

Free listing was implemented with a convenience sample of IDPs, Veterans and family members in Kharkiv and Zaporizhia. In addition to the standard DIME questions, we asked one additional question: “please list all the features of a mental health service that people in your community would want to go to.”

Three FGDs were conducted – two with IDPs and one with Veterans. During the focus groups, results from the FLs were presented and participants were asked: 1) their opinions of the results; 2) whether there was anything to add; 3) what makes people feel satisfied or dissatisfied with different types of mental health services; and 4) what would influence their decisions to attend mental health services. Draft versions of the vignettes created by the JHU and local research team with input from the FL data, were also presented, and participants were asked to discuss their reactions to the vignettes, as well as important aspects that might be missing from these depictions.

### Instrument adaptation

The development of the AMHR implementation science measure for consumers (the basis for adaptation and testing) is described in detail elsewhere [12, 13]. Adaptation consisted of adding items based on the qualitative data and incorporating phrases used by participants during qualitative interviews in the wording of individual items. As the AMHR measure was originally designed for post program use, we also adjusted the wording of response options, by re-wording questions in the hypothetical (e.g. instead of “the program fits with my cultural beliefs” we used “the program *would* fit with my cultural beliefs”) for use with the vignettes.

### Vignette development

The validation process required the development of two vignettes: one depicting a well-implemented mental health program and one depicting a “poorly”-implemented mental health program. Based on initial stakeholder meetings, feedback from local collaborators, and the qualitative study, it became clear that the standard of care in Ukraine (one that is heavily based on inpatient psychiatric services) was considered “poorly”-implemented for our target population. While psychiatric care is well established in Ukraine, it was considered unresponsive and not accessible to the population we are working with. As such, existing psychiatric care became the basis for the “poorly”-implemented vignette (Additional file 4). To develop a hypothetical well-implemented program vignette we drew on formative work in Ukraine, current global mental health research, and AMHR’s prior research and programmatic experience in other countries. This hypothetical vignette described a community-based psychotherapy approach. The local PI (BD), who was familiar with standard of care in Ukraine wrote the “poorly”-implemented vignette, and other authors (EH & PB) wrote the hypothetical community-based vignette (Additional file 3). Additional adjustments and adaptations were made based on key concepts that emerged from FL and FGDs (described below). The vignettes were translated into Russian and back-translated into English. While Ukrainian is the national language of Ukraine, Russian is widely spoken, especially in Eastern and Central Ukraine. Using the vignettes, we then pilot tested the measure with our local research team to help ensure that there was enough detail in the vignettes to be able to respond to all items on the measure.

#### Quantitative study to test the reliability and validity of adapted implementation measures

### Measures

The AMHR consumer implementation measure includes subscales designed to measure the four implementation constructs: *Acceptability* (17 items); *Appropriateness* (13 items); *Feasibility* (14 items) and *Accessibility* (8 items). Each item was scored on a four point Likert-type scale with response options 0 “Not at all,” 1 “A little bit,” 2 “A moderate amount,” and 3 “A lot.” We also included a response option “don’t know” in case there was not enough information in the vignettes to answer the item or participants felt like they could not otherwise respond. The measure used in the study is included in Additional file 5 and is available on request through the AMHR website (https://www.jhsph.edu/research/centers-and-institutes/global-mental-health/).

### Participants

Potential participants were recruited by local organizations serving the study population (IDPs, veterans). Staff at these organizations approached individuals using an information sheet describing the study. If potential participants were interested and agreed to be contacted by the study team, staff passed along their contact information to study staff. As part of the larger validity study, participants met with psychiatrist and psychotherapist interviewers, gave consent, were screened for eligibility and ultimately self-administered an assessment battery (including both mental health symptom scales and the AMHR implementation measure) on a handheld device. To be eligible, participants had to be over 18 and either internally displaced and/or a veteran of the Ukraine military. Exclusion criteria consisted of a) active psychosis; b) affected by a major developmental delay; and/or c) currently a danger to themselves or others.

### Research design

Using an experimental vignette study design [19, 20], we randomly assigned half the participants to read and respond to the “poorly”-implemented program, while the other half read and responded to the “well”-implemented program vignette. We hypothesized that scores across all sub-scales on the Implementation measure would be higher for the “well”-implemented compared to the “poorly”-implemented vignette. At the end of the interview, participants were provided explanations of each of the scale domains (acceptability, appropriateness, feasibleness, and accessibility) and asked to provide overall ratings of the program depicted in the vignette on each of these domains. Overall summary ratings were based on a 0 to 3 scale from “no, not at all *acceptable/appropriate/feasible/accessible*” to “yes, *acceptable/appropriate/feasible/accessible*.” The domain explanations were based on Proctor et al. outcomes for implementation research [1] which we had previously operationalized for use in non-Western settings [13, 21].

### Analysis

Scores for each domain were generated by calculating the mean response across all items on each sub-scale. Test-retest reliability was assessed by repeat administration of the same vignette and instrument 2–3 days later to a randomly selected sub-group (approximately one-third of the sample), calculating Spearman’s Rank Order correlation coefficient (rho) for each scale. Scores above *rho* of 0.7 are acceptable, while scores above *rho* 0.8 are considered good. Internal consistency reliability, using Cronbach’s Alpha, was also calculated [22].

We examined construct validity by determining the degree to which each scale is associated with other scales. Based on conceptual frameworks [23] that show these constructs are important to implementation we would expect them to be correlated, but not perfectly [1]. Finally, in the absence of a gold standard, criterion validity would be supported if the average scores across the scales were significantly higher when participants responded to the questions based on reading the well-implemented program vignette compared to responding to questions based on reading the “poorly”-implemented program vignette. As part of criterion validity, we also examined the association of scale scores to overall summary ratings of the assigned vignette. We hypothesized that higher scores on the measures would be associated with higher overall domain ratings of the program depicted in the vignette. We dichotomized overall domain ratings yielding an odds ratio representing the increased odds of having rated the vignette as a moderate amount or very *acceptable/appropriate/feasible/accessible* compared to a little bit or not at all *acceptable/appropriate/feasible/accessible*.

### Missing data

We expected a large amount of missing data based on asking participants to answer hypothetically about their assessments of a program not yet in existence. While efforts (i.e. pilot testing of instruments with vignettes by study staff and local investigator) were taken to ensure there was enough information in the vignettes to answer each question, some participants still marked “don’t know.” For internal consistency reliability and factor analyses we included any complete item level data. For analyses based on summary scores we pro-rated the scale scores only for participants who responded to more than 50% of each scale by replacing missing items with the individual average score across the items in each domain. For example, for appropriateness, only participants who provided responses to 7 or more items were included in the analysis of the appropriateness scale (but their data was included in analyses of other domains if they provided more complete answers to those scales).

## Results

### Qualitative study

#### Participants

In total, *N =* 169 male and female participants (ages 18–77) in Kharkiv and Zaporizhia took part in the qualitative study. One hundred and twenty-four participants provided free lists and 46 participants participated in focus group discussions. Participants included IDPs and veterans of the Ukraine military anti-terrorist operation.

#### Results of free listing

Fifty-three different aspects of mental health programming that would affect whether he/she would want to attend the program were mentioned by two or more participants. The most commonly mentioned aspects were “Professionalism/Competencies of the Provider” (*n =* 39; 35%) and “being informed about the existence of the program” (*n =* 34; 27%) (Table 1).

**Table 1:** *Aspects of mental health programs that would make participants want to attend* (*n =* 124)

	*N*	%
1. Professionalism/Qualifications/Competence	39	31.5%
2. Being informed about available services	34	27.4%
3. Convenience, accessibility	27	21.8%
4. Work with all ages and groups	24	19.4%
5. Friendly, trusting atmosphere / companionship/ pleasant communication	23	18.5%
6. Really helps/Effectiveness	21	16.9%
7. Help from someone you trust	21	16.9%
8. Support, warm-heartedness, tenderness	19	15.3%
9. Interest	17	13.7%
10. Confidentiality	17	13.7%

#### Results of focus group discussions

During the FGDs, common themes emerged including: a) the educational/professional background of the clinician must be strong; b) the costs and wait times associated with services are often too high; c) services and providers need to be accessible (and often are not); d) the location and atmosphere of where services are delivered is important; and e) concerns about confidentiality and stigma associated with receiving mental health treatments. For example, when discussing psychotherapists one participant stated that a “normal psychotherapist with [just] any psychological education will not be able to deal with the stress of clients,” while another participant expressed concerns about psychiatric help saying “waiting time is too long. You may meet the psychiatrist one time and not see them again until a few weeks later.” Others were concerned that psychiatric treatment resulted in being registered into a database that could be used against them. The information from these discussions allowed us to add content to both the vignettes and the measures.

#### Adaptation of measures and vignettes

##### Measures

The most significant adaptation to the measure was in re-wording the questions so that they could be asked in the hypothetical. For example, we reworded the question “Did you feel satisfied with the services you received?” to “Would you feel satisfied with receiving the services depicted in the story?” Items on the existing measures covered many of the concerns that arose during FL and FGDs, but three additional items were added based qualitative data. These included: “Would you feel like your counselor would take an interest in you?” (Acceptability) “How convenient would it be to get to the place where you would meet your provider?” and “Do you think the place where you would meet your provider would be comfortable?” (Feasibility). No items were removed.

##### Vignettes

Results from the FL and FGDs were used to add content and language to the vignettes. For example, due to the frequency in which provider qualification/experiences arose as concerns in the qualitative study, we made sure to clearly describe the educational and experiential background of the providers in both vignettes. In the “poorly”-implemented program vignette describing existing psychiatric services providers were described as having a medical degree with various years of experience (5–10 years). In the “well”-implemented program vignette we stated: “Counselors would mostly be psychologists, social workers or other people who currently work with trauma-affected populations including veterans, their families and IDPs. Most counselors would have a background in psychology, but not all.” We also provided descriptions in each vignette about how people would hear about the services. The “poorly”-implemented vignette stated that people would hear about services “through doctors and the health care system.” We described people hearing about the “well”-implemented service through organizations, advertisements, word of mouth and social media, based on data from the FL suggesting that people would prefer to find out about programs from these sources and that this would promote care-seeking.

### Quantitative study

### Participants

A total of *N =* 153 participants (*n =* 109 in Zaporizhia, *n =* 44 in Kyiv) were interviewed. The majority of participants were male (54%) and married (56%). Overall, the sample was highly educated with over half of participants (58%) having received at least a university degree (Table 2).

**Table 2:** *Sample Characteristics*

	*N =* 153	
	*n*	*%*
Mean Age (SD)	39 (11)	
Site		
Kyiv	44	29
Zaporizhia	109	71
Male	83	54
Marital status		
Single	31	20
Married	86	56
Widowed	9	6
Divorced	27	18
Education		
Primary	4	3
High School	18	12
Vocational	42	27
University	83	54
Post-university	6	4

#### Missing data

Missing data was common (range: 6.5 to 66.7% missing). We hypothesize that this was largely due to people not having enough information in the vignettes to answer the specific questions. The item with the largest percentage of missing data was “How much would receiving the mental health services described affect your income?”, a *feasibility* question. No demographic variables were associated with high levels of missing variables on a scale. Fifteen participants were removed from the acceptability analysis; 28 from appropriate, 8 from feasibility, and 5 from the accessibility analysis.

#### Reliability

Internal consistency reliabilities were good for *acceptability*, *feasibility*, and *accessibility* (*α* = 0.89; *α* = 0.85; and *α* = 0.85 respectively), and excellent for *appropriateness* (*α* = 0.91). A random selection of *n =* 27 participants contributed data to test-retest reliability calculations (*n =* 10 from Kyiv; *n =* 17 from Zaporhizia). The average time between interviews was 4.2 days (*SD =* 2.0; Range: 2–9 days). Test-rest reliability was low for *accessibility* (*rho =* 0.61), and good for *acceptability*, *appropriateness*, and *feasibility* (*rho =* 0.70*; rho =* 0.79; and *rho =* 0.76 respectively).

#### Validity

##### Construct validity

Scale scores across domains were significantly associated with scores on other domains. The lowest correlation was between total scores on the *acceptability* scale and the *accessibility* scale (*rho =* 0.39, *p <* 0.05), while the highest correlation was between *appropriateness* and *feasibility* (*rho =* 0.62; *p <* 0.05) (Results not shown).

##### Criterion validity

**Fig. 2:**
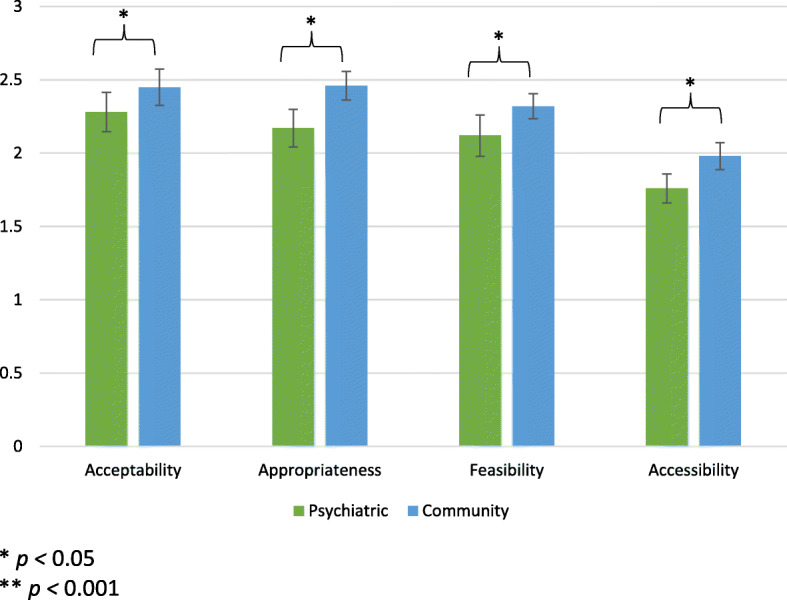
Average scale scores by vignette

We were interested to see whether our scales could differentiate between a “well”-implemented program and a “poorly”-implemented program, good implementation being based on qualitative data from this population, stakeholder feedback, and our own experience in other countries. Our hypothesis was that people who responded to the “well”-implemented vignette would have substantially higher average scores across all domain scales compared to those who responded to the “poorly”-implemented vignette. Most scales demonstrated substantial and statistically significant differences in average scores by vignette assignment. Each unit increase in scores on the scales was associated with higher odds of having been randomly assigned and responded to the “well”-implemented compared to the “poorly”-implemented vignette. Odds ratios were highest for *appropriateness* and *feasibility* suggesting substantially different scores on these measures for those who rated the “well”-implemented service compared to those rating the “poorly”-implemented service (Table 3; Fig. 2).

**Table 3:** *Associations between scale scores and type of vignette*

		**Logistic Regression** ^a^		
	*N*	OR	95% CI	*p-value*
Acceptability	136	2.21	[1.05, 4.70]	0.038*
Appropriateness	110	5.60	[1.88, 16.71]	0.002*
Feasibility	122	4.41	[1.57, 12.42]	0.005*
Accessibility	103	3.39	[1.25, 9.20]	0.017*

^a^0 = not acceptable/appropriate/feasible/accessible; 1 = yes acceptable/appropriate/feasible/accessible

* *p <* 0.05

**Fig. 3:**
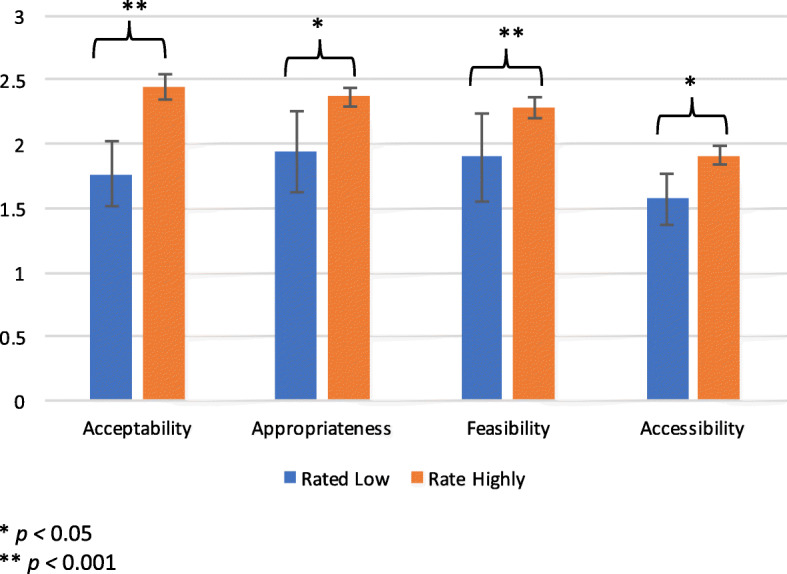
Average scale scores by overall rating of the program depicted in the vignette

We also compared scores on each scale to the respondent’s own overall rating on each domain (Table 4; Fig. 3). Higher scale scores on all domains were significantly associated with better overall summary ratings on that domain. For example, each unit increase on the *acceptability* scale was associated with a 13-times higher odds of rating the program depicted in the vignette as *acceptable* compared to *unacceptable*.

**Table 4:** *Associations between scale scores and overall summary ratings of the program depicted in the vignette*

		**Logistic Regression** ^a^		
	*N*	OR	95% CI	*p-value*
Acceptability	135	12.98	[3.80, 44.4]	0.000**
Appropriateness	109	5.10	[1.65, 15.79]	0.005*
Feasibility	121	14.47	[3.67, 57.16]	0.000**
Accessibility	102	5.75	[1.49, 22.24]	0.011*

^a^0 = not acceptable/appropriate/feasible/accessible; 1 = yes acceptable/appropriate/feasible/accessible

* *p <* 0.05

** *p <* 0.001

## Discussion

A significant body of research has been published stating the unmet need for improving psychometric qualities of Implementation measures [3, 11, 24–26]. In HIC, there are some strong starting points such as frameworks [11], individual construct measures with strong psychometric support (e.g. leadership, [27] organizational readiness for change [28], and compendiums of implementation measures [24, 25]. Despite a new focus on implementation science in global mental health (GMH) research, there are no validated GMH measures for Implementation Science outcomes in LMIC. Our team’s initial work followed the process outlined in Martinez et al. [11], using home-grown instruments and starting with a shorter, practical measure (e.g., multi-construct, 10 items or less for each construct). While attempting to psychometrically evaluate our initial implementation measure, we encountered significant challenges in LMIC due to an overall lack of mental health services and systems [6]. Most implementation research respondents in LMIC, particularly at consumer level or when working with community-based providers and organizations, lack the context (e.g. little exposure, limited experience participating in services) of a mental health system including the variety of possible types of services that may exist. Without this knowledge, there is little basis for generating opinions on the quality and type of implementation. In other words, it is hard to rate the “relative advantage” [29] of a service or its implementation if there are no existing services with which to make a comparison.

Given this, we needed a different method to test and validate implementation measures prior to their use. We employed an experimental vignette study design [19], one that has been used in other studies to test implementation measures in the United States [17]. We first collected local qualitative data to inform both the local adaptation of the instruments and the development of the vignettes, then tested them using qualitative methods. Use of mixed-methods research to develop and validate measures is not new in global mental health [21, 30–34] yet has rarely been done with implementation science measures, even in the US [11, 35]. Use of these methods could provide a widely generalizable process to develop locally relevant and valid implementation measures to facilitate global mental health implementation science research.

Results indicated the *acceptability*, *appropriateness*, *feasibility*, and *accessibility* scales were generally reliable and valid. Scores on the scales were significantly higher for those rating the “well”-implemented program (i.e. psychotherapy vignette) compared to the “poorly”-implemented program. Local content validity was supported: when participants were presented with an explanation of the meaning of each domain and asked to rate the vignette program overall, scale scores were significantly associated with higher ratings compared to lower ratings.

Our *accessibility* scale performed less well, with low test-retest reliability. The poor reliability of the accessibility scale suggests problems with consistency of responses. The *accessibility* scale required participants to provide their opinions on whether the program would be accessible to other subgroups of people in the population (e.g. women, men, etc.). Commenting on the accessibility of the program for others may be challenging for participants to do reliably. When administering these scales in the future, researchers should consider whether respondents are knowledgeable enough about the question being asked to provide an informed response to the items – a challenge for global mental health research where exposure to a range of types of services is limited.

We used the vignette based approach as a way to allow potential consumers to comment on what they would want and not want in a mental health care program. In other words, the vignettes provided a way for people to provide their input on the implementation of mental health programs without ever having to have actually experienced these programs directly. While this approach may not be necessary in contexts where participants have familiarity with a range of services, use of vignettes was a practical tool to test the measures prior to their use among participants who lack familiarity with a variety of services and range in quality of implementation. Other methods of validation in similar contexts might include only comparing to overall ratings (as in our study) and a variation on known group validation, whereby consumers of programs that are “well”-implemented rate the program and these responses are compared to responses of consumers who attend a “less-well”-implemented program.

This study is the first to assess the validity of measures for the major implementation science domains among potential mental health service consumers in global mental health settings. Consumers are often included in the major theoretical frameworks that guide current D&I research [1, 14, 16], and are considered key stakeholders in the implementation process [36, 37]. However, few if any, measurement instruments exist to study implementation domains from the consumer perspective. Our work in developing instruments that would capture this perspective is unique.

### Strengths and limitations

Strengths of the study include a robust sample size from multiple regions in Ukraine, qualitative methods to enhance adaptations, and use of a generalizable method and experimental vignette design to test implementation measures prior to use. Limitations include a significant amount of missing data, likely due to not enough information included in the vignettes, and unknown generalizability of the findings to other settings. Future studies should include more extensive pilot testing of the vignettes and instruments among a small sample of participants with the aim of reducing missing data. Regarding the AMHR implementation measure: local adaptation and testing of this measure is warranted before being used in other settings.

## Conclusions

Advancing and strengthening measurement approaches for D&I research is critical to building a cumulative scientific knowledge base. This study describes the use of a novel approach to testing the reliability and validity of implementation science measure examining multiple D&I constructs in global mental health research. Building on our previous work developing mental health measures in LMIC and work being done in the United States to validate implementation measures, we incorporated key qualitative data to help ensure local relevance and ecologic validity. Our psychometric process performed well and suggested that most scales were reliable and valid for potential mental health service consumers in Ukraine. The results of this research illustrate a potentially useful method to assess the psychometrics of implementation measures in global mental health research. More testing is needed to demonstrate the validity of these measures in other LMIC settings.

## Additional files

Additional file 1: Adult Qualitative_free list. *Qualitative guide used for free listing activity*. (DOC 46 kb)

Additional file 2: Adult Qualitative_FGD Guide. *Qualitative guide used for focus group activity*. (DOCX 17 kb)

Additional file 3: D&I Assessment Tool. *Quantitative tool for dissemination and implementation questions including the vignette based on community mental health services in English*. (DOCX 40 kb)

Additional file 4: Consumer_Psychiatric Vignette. *Vignette depicting current standard of care in Ukraine*. (DOCX 13 kb)

Additional file 5: UkraineConsumerAssessmentTool_ FieldReady _ENG. *Full quantitative assessment used in the study*. (DOCX 114 kb)

## Abbreviations

AMHR: Applied Mental Health Research Group
CFIR: Consolidated Framework for Implementation Research
D&I: Dissemination and Implementation
EPIS: Exploration, Preparation, Implementation and Sustainment framework
FGD: Focus Group Discussions
FL: Free Listing
GMH: Global Mental Health
HIC: High Income Countries
IDP: Internally Displaced Persons
LMIC: Low- and Middle-Income Countries
NGO: Non-Governmental Organizations
RE-AIM: Reach, Effectiveness, Adoption, Implementation and Maintenance framework
VOT: United States Agency for International Development Victims of Torture Fund
