# Supplementary material for: Measuring implementation in global mental health: validation of a pragmatic implementation science measure in eastern Ukraine using an experimental vignette design
Source: BMC Health Serv Res. 2019 Apr 29;19:262. doi: 10.1186/s12913-019-4097-y (PMC6489318; doi:10.1186/s12913-019-4097-y)
Supplement: Supplementary file 1 — Adult Qualitative_free list. Qualitative guide used for free listing activity. (DOC 46 kb) [file 12913_2019_4097_MOESM1_ESM.doc]

Aim to interview someone who has come from the conflict area because of the conflict (IDP) or a person who has served in the Ukrainian military.

Interviewer ID #: _____ Interviewer or Note taker #:________________

Respondent: Age (years) : _____ Sex : _____ Marital Status : ______________

**1. Primary Question**

What are all of the problems facing (IDP/Veteran) adults?

Problem Brief Description (1-2 phrases)

1. ________________________ _____________________________________________________________________________

_____________________________________________________________________________

2. ________________________ _____________________________________________________________________________

_____________________________________________________________________________

3. ________________________ _____________________________________________________________________________

_____________________________________________________________________________

4. ________________________ _____________________________________________________________________________

_____________________________________________________________________________

5. ________________________ _____________________________________________________________________________

_____________________________________________________________________________

6. ________________________ _____________________________________________________________________________

_____________________________________________________________________________

7. ________________________ _____________________________________________________________________________

_____________________________________________________________________________

8. ________________________ _____________________________________________________________________________

_____________________________________________________________________________

9. ________________________ _____________________________________________________________________________

_____________________________________________________________________________

10. ________________________ _____________________________________________________________________________

2. **Primary Question**

What are all of the problems facing families of (IDP/Veterans)?

Problem Brief Description (1-2 phrases)

1. ________________________ _____________________________________________________________________________

_____________________________________________________________________________

2. ________________________ _____________________________________________________________________________

_____________________________________________________________________________

3. ________________________ _____________________________________________________________________________

_____________________________________________________________________________

4. ________________________ _____________________________________________________________________________

_____________________________________________________________________________

5. ________________________ _____________________________________________________________________________

_____________________________________________________________________________

6. ________________________ _____________________________________________________________________________

_____________________________________________________________________________

7. ________________________ _____________________________________________________________________________

_____________________________________________________________________________

8. ________________________ _____________________________________________________________________________

_____________________________________________________________________________

9. ________________________ _____________________________________________________________________________

_____________________________________________________________________________

10. ________________________ _____________________________________________________________________________

_____________________________________________________________________________

3. **Primary Question**

What are all of the problems facing children of (IDP/Veterans)?

Problem Brief Description (1-2 phrases)

1. ________________________ _____________________________________________________________________________

_____________________________________________________________________________

2. ________________________ _____________________________________________________________________________

_____________________________________________________________________________

3. ________________________ _____________________________________________________________________________

_____________________________________________________________________________

4. ________________________ _____________________________________________________________________________

_____________________________________________________________________________

5. ________________________ _____________________________________________________________________________

_____________________________________________________________________________

6. ________________________ _____________________________________________________________________________

_____________________________________________________________________________

7. ________________________ _____________________________________________________________________________

_____________________________________________________________________________

8. ________________________ _____________________________________________________________________________

_____________________________________________________________________________

9. ________________________ _____________________________________________________________________________

_____________________________________________________________________________

10. ________________________ _____________________________________________________________________________

_____________________________________________________________________________

Note to interviewer: Ask men about activities for men; ask women about activities for women.

4. **Primary Question**

Please list all of the activities that (IDPs/Veterans) perform to care for themselves:

Activities Brief Description (1-2 phrases)

1. ________________________ _____________________________________________________________________________

_____________________________________________________________________________

2. ________________________ _____________________________________________________________________________

_____________________________________________________________________________

3. ________________________ _____________________________________________________________________________

_____________________________________________________________________________

4. ________________________ _____________________________________________________________________________

_____________________________________________________________________________

5. ________________________ _____________________________________________________________________________

_____________________________________________________________________________

6. ________________________ _____________________________________________________________________________

_____________________________________________________________________________

7. ________________________ _____________________________________________________________________________

_____________________________________________________________________________

8. ________________________ _____________________________________________________________________________

_____________________________________________________________________________

9. ________________________ _____________________________________________________________________________

_____________________________________________________________________________

10. ________________________ _____________________________________________________________________________

_____________________________________________________________________________

Note to interviewer: Ask men about activities for men; ask women about activities for women.

**5. Primary Question**

Please list all of the activities that (IDPs/Veterans) perform to care for their families:

Activities Brief Description (1-2 phrases)

1. ________________________ _____________________________________________________________________________

_____________________________________________________________________________

2. ________________________ _____________________________________________________________________________

_____________________________________________________________________________

3. ________________________ _____________________________________________________________________________

_____________________________________________________________________________

4. ________________________ _____________________________________________________________________________

_____________________________________________________________________________

5. ________________________ _____________________________________________________________________________

_____________________________________________________________________________

6. ________________________ _____________________________________________________________________________

_____________________________________________________________________________

7. ________________________ _____________________________________________________________________________

_____________________________________________________________________________

8. ________________________ _____________________________________________________________________________

_____________________________________________________________________________

9. ________________________ _____________________________________________________________________________

_____________________________________________________________________________

10. ________________________ _____________________________________________________________________________

_____________________________________________________________________________

**6. Primary Question**

Please list all the aspects of a mental health service that people in your community would go to?

Aspects Brief Description (1-2 phrases)

1. ________________________ _____________________________________________________________________________

_____________________________________________________________________________

2. ________________________ _____________________________________________________________________________

_____________________________________________________________________________

3. ________________________ _____________________________________________________________________________

_____________________________________________________________________________

4. ________________________ _____________________________________________________________________________

_____________________________________________________________________________

5. ________________________ _____________________________________________________________________________

_____________________________________________________________________________

6. ________________________ _____________________________________________________________________________

_____________________________________________________________________________

7. ________________________ _____________________________________________________________________________

_____________________________________________________________________________

8. ________________________ _____________________________________________________________________________

_____________________________________________________________________________

9. ________________________ _____________________________________________________________________________

_____________________________________________________________________________

10. ________________________ _____________________________________________________________________________

_____________________________________________________________________________

Additional probes for question 6:

- What would make people feel satisfied with the service?
- What would make people feel unsatisfied with the service?
- How would this service help people?
- What would make people continue to go to the service?
- What would make people stop using the service?
- What would be all the things that make it difficult for people to attend this service?
- What types of arrangements would people have to make to attend this service?
