# Supplementary material for: Measuring implementation in global mental health: validation of a pragmatic implementation science measure in eastern Ukraine using an experimental vignette design
Source: BMC Health Serv Res. 2019 Apr 29;19:262. doi: 10.1186/s12913-019-4097-y (PMC6489318; doi:10.1186/s12913-019-4097-y)
Supplement: Supplementary file 2 — Adult Qualitative_FGD Guide. Qualitative guide used for focus group activity. (DOCX 17 kb) [file 12913_2019_4097_MOESM2_ESM.docx]

**Ukraine Qualitative Study/ Phase 1**

**Focus Group Discussion Guide for the Community (Adult IDPs and Veterans)**

***Instructions:***

*Work in pairs. One person acts as moderator and the other takes detailed notes. After obtaining consent from each participant, privately, and moving to a private location for the focus group discussion:*

*Greet all participants*

*In your notebooks fill in information about date and site of interview, name of interviewers, number of interviewees and their ages and gender. The note-taker should create a numbered seating chart in the note-book to aid with note-taking.*

***Opening***

1. We are interested in learning about mental health services in your community. Can you tell us what you know about this?

*[Probes]*
Where can people in your community go to get help for mental health problems?

What kinds of help are available?

Who provides this help?

What do you think of mental health services?
How are they helpful? Not helpful?

What aspects of these services are good? Not good?

What changes do you think should be made to existing mental health services to improve them?

***Prompt: Responses to service implementation questions from interviews***

1. Earlier, we spoke with other people in your community about mental health services that they would consider going to if they needed help with a problem. They told us what they thought of different services. We would like to learn more about this by asking you to respond to some of what they told us and to share what you think.

*[For each topic, provide a summary of information from free list and key informant interviews and probe for additional information]*

*For each, ask:* What do you think about this? What, if any, aspects are missing?

- Which problems does *[Name specific type of service]* help with?
- Describe problems that are not being addressed by *[Name specific type of service]*?
- Reasons to go to *[Name specific type of service]* for help
- Reasons not to go to *[Name specific type of service]* for help
- What makes it difficult to get help from *[Name specific type of service]*?
- What makes it easy to get help from *[Name specific type of service]*?
- What would make people feel satisfied with *[Name specific type of service]*
- What would make people feel dissatisfied with *[Name specific type of service]*
- What would make people choose to continue getting help from *[Name specific type of service]*
- What would make people choose to stop getting help from *[Name specific type of service]*

***Prompt: Responses to function questions from interviews***

Now we want to move onto a different topic. We also spoke with people in your community about what IDPs/ Veterans *[read appropriate category]* do to take of themselves and their families. We would like to learn more about this by talking with you as well. We are going to share some of what people told us during our earlier interviews and would like to know what you think about this.

1. Many people said that the activities that IDPs/ veterans *[read appropriate category]* do to take care of themselves are *[Read selected activities/ tasks].*  What do you think about this?

*[Probes]*

Can you tell us about any tasks or activities that you think are missing?
Can you prioritize these activities in order of what most people in the community think is important?

1. Many people said that the activities that IDPs/ veterans *[read appropriate category]* do to take care of their families are *[Read selected activities/ tasks].*  What do you think about this?

*[Probes]*

Can you tell us about any tasks or activities that you think are missing?
Can you prioritize these activities in order of what most people in the community think is important?

***Closing***

1. Is there anything else you think we should know or that you would like to say?
2. Do you have any questions for us?

At the end of the discussion the note-taker should review the notes while the group is still present. If anything is not clear, ask for clarification and correct the notes as necessary.

**Thank you.**
