# Supplementary material for: Measuring implementation in global mental health: validation of a pragmatic implementation science measure in eastern Ukraine using an experimental vignette design
Source: BMC Health Serv Res. 2019 Apr 29;19:262. doi: 10.1186/s12913-019-4097-y (PMC6489318; doi:10.1186/s12913-019-4097-y)
Supplement: Supplementary file 3 — D&I Assessment Tool. Quantitative tool for dissemination and implementation questions including the vignette based on community mental health services in English. (DOCX 40 kb) [file 12913_2019_4097_MOESM3_ESM.docx]

**Dissemination and Implementation Assessment Tool**

Now we are going to switch the focus of the questions. For the following questions, please read the description of the mental health program and then answer the questions based on your opinions. If you do not have an opinion about the question or do not know, please indicate that.

**Mental health program**

This service is to assist persons with psychological problems due to trauma that are severe enough to affect their ability to function as individuals and as members of their family and society. The program is not for severe mental illness that needs a psychiatrist, hospital, and drugs. It is for the types of problems that everyone may get at some stage in their lives, particularly people who have been through a lot of stress and trauma. This includes problems like depression, anxiety, alcohol and drug overuse, and stress related problems. Some people get these problems worse than others and need help to get over them, which is what this service would provide.

The service would focus on Internally Displaced Persons (IDPs) and veterans and their families. It is a counseling or talk‐based program. Each week the counselor meets with you for an hour (or longer if needed) to discuss ongoing problems and skills that can help address these problems. It is a time‐limited program lasting 5‐12 sessions. These would usually be provided weekly although frequency could vary between 2/week to 1 every 2 weeks, depending on client and counselor availability. You would need to get yourself to and from the session with the counselor or arrange that the counselor meets you in a location where you feel comfortable. In each session, your counselor would do an activity related to your thoughts, feelings or behaviors. For example, you might talk about unhelpful thoughts you have such as “I am no good” and work together to change that to a more helpful thought like “I am not great at everything but I am a very trustworthy friend.” After each session you would also have things to practice before the next meeting (i.e. homework). The time practice takes is very different for each person – some choose to practice a skill each day while others have limited time and would only spend about 15 minutes on it throughout the week. There is no drug treatment associated with the service. There are also no goods provided – such as money or food. Improving symptoms/problems and functioning is done entirely through talk, the relationship with the counselor, and practicing new skills during weekly sessions and at home.

Your counselor would be available to you on a weekly basis, during your session. If an emergency came up, your counselor or their supervisor would be available by phone as well. Your meetings with your counselor would be held in a private space where you feel comfortable. This could include meeting at formal health clinics, private offices, living spaces, community centers, schools or other accessible places, and in the home for some sessions if necessary. A requirement of all meeting locations would be that they provide private space so that your sessions with your counselor would not be overheard.

Counselors would mostly be psychologists, social workers or other people who currently work with trauma‐affected populations including veterans, their families and IDPs. Most counselors would have a background in psychology, but not all. Counselors may work for organizations, government agencies or be self‐employed. Counselors are trained in how to recognize signs and symptoms of common problems related to thinking and feeling, how to teach helpful skills to relieve these types of problems and how to maintain confidentiality (i.e. keep the information you tell them private).

In order for the service to continue and be used, people would have to know about it. So organizations and counselors who are providing the program might advertise it through print media (e.g. paper flyers, information sheets), through word of mouth, or through other types of media (e.g. social networking sites).

| **2.1 Adoption** | | | | | | |
| --- | --- | --- | --- | --- | --- | --- |
|  |  |  | | | | |
| AD01 | What would make you want to participate in this type of mental health program? |  | | | | |
|  |  |  | | | | |
| AD02 | What would make you stop participating in this type of program if you had already started? |  | | | | |
|  | | 0  Not at all | 1  A little bit | 2  A moderate amount | 3  A lot | 8  Don’t know |
| AD03 | Would you discuss with others (e.g. family, friends, coworkers, or any other people) about what the mental health program is? | 0 | 1 | 2 | 3 | 8 |
| AD04 | Would you discuss with others (e.g. family, friends, coworkers, or any other people) what you need to do if you participate in the mental health program? | 0 | 1 | 2 | 3 | 8 |
|  |  |  |  |  |  |  |
| AD05 | Would you use the skills you learned in the mental health program in the future? | 0 | 1 | 2 | 3 | 8 |
|  |  |  |  |  |  |  |
| AD06 | Would you encourage others to seek the mental health program’s services? | 0 | 1 | 2 | 3 | 8 |
|  |  |  |  |  |  |  |
| AD07 | Would you refer others with similar problems to the mental health program? | 0 | 1 | 2 | 3 | 8 |
|  |  |  |  |  |  |  |
| AD08 | Would you to return to the mental health services if you felt like you needed them in the future? | 0 | 1 | 2 | 3 | 8 |

# *removed original items 11 and 12 because they are redundant with our wording for existing items 5 and 6

|  | | | | | | |
| --- | --- | --- | --- | --- | --- | --- |
| **2.2 Acceptability** | | | | | | |
|  | | 0  Not at all | 1  A little bit | 2  A moderate amount | 3  A lot | 8  Don’t know |
| AC01 | Overall, do you like the mental health program described? | 0 | 1 | 2 | 3 | 8 |
| AC02 | Would you like attending the mental health treatment sessions as part of the program? | 0 | 1 | 2 | 3 | 8 |
| AC03 | Would you feel satisfied with the mental health treatment in the program described? | 0 | 1 | 2 | 3 | 8 |
|  |  |  |  |  |  |  |
| AC04 | Would you enjoy learning the mental health program described? | 0 | 1 | 2 | 3 | 8 |
|  |  |  |  |  |  |  |
| AC05 | Would you feel like the skills you learned in the mental health program are useful? | 0 | 1 | 2 | 3 | 8 |
|  |  |  |  |  |  |  |
| AC06 | Do you feel like the components (i.e. the strategies you learn in session) of the mental health program make sense? | 0 | 1 | 2 | 3 | 8 |
|  |  |  |  |  |  |  |
| AC07 | Would you feel comfortable raising questions to your counselor if you participated in the mental health program? | 0 | 1 | 2 | 3 | 8 |
|  |  |  |  |  |  |  |
| AC08 | Would you feel that the counselor in the mental health program would listen to your concerns and questions about the program? | 0 | 1 | 2 | 3 | 8 |
|  |  |  |  |  |  |  |
| AC09 | Would you feel satisfied with your counselor’s abilities in the mental health program? | 0 | 1 | 2 | 3 | 8 |
|  |  |  |  |  |  |  |
| AC10 | Would you feel that your counselor would address any of your questions or concerns about the program? | 0 | 1 | 2 | 3 | 8 |
|  |  |  |  |  |  |  |

| AC11 | Would you feel that your counselor was available when you needed to talk to him/her? | 0 | 1 | 2 | 3 | 8 |
| --- | --- | --- | --- | --- | --- | --- |
|  |  |  |  |  |  |  |
| AC12 | Would you feel that you could trust your counselor? | 0 | 1 | 2 | 3 | 8 |

*Remove original item 13 as not able to test in validity study

|  | | | | | | |
| --- | --- | --- | --- | --- | --- | --- |
| **2.3 Appropriateness** | | | | | | |
|  | | 0  Not at all | 1  A little bit | 2  A moderate amount | 3  A lot | 8  Don’t know |
| AP01 | How well does the mental health program described fit with your cultural values? | 0 | 1 | 2 | 3 | 8 |
| AP02 | How well does the mental health program described fits with your personal values? | 0 | 1 | 2 | 3 | 8 |
| AP03 | How well would participating in the mental health program fit into your daily schedule and routine? | 0 | 1 | 2 | 3 | 8 |
|  |  |  |  |  |  |  |
| AP04 | How well does the mental health program described fit with the male culture in your country? | 0 | 1 | 2 | 3 | 8 |
|  |  |  |  |  |  |  |
| AP05 | How well does the mental health program described fit with the female culture in your country? | 0 | 1 | 2 | 3 | 8 |
|  |  |  |  |  |  |  |
| AP06 | Do you feel that the organization(s) providing the mental health program is a good place for delivery of these services? | 0 | 1 | 2 | 3 | 8 |
|  |  |  |  |  |  |  |
| AP07 | Would you feel comfortable with the location where you would meet a counselor for this program? | 0 | 1 | 2 | 3 | 8 |
|  |  |  |  |  |  |  |
| AP08 | Do you think the mental health program would be a good way to address your problems? | 0 | 1 | 2 | 3 | 8 |
|  |  |  |  |  |  |  |
| AP09 | Do you think you would learn helpful strategies from the program to deal with your problematic thoughts, feelings and behaviors? | 0 | 1 | 2 | 3 | 8 |
|  |  |  |  |  |  |  |

| AP10 | Do you think the mental health program would help you with your problems? | 0 | 1 | 2 | 3 | 8 |
| --- | --- | --- | --- | --- | --- | --- |
|  |  |  |  |  |  |  |
| AP11 | Do you think the mental health program would be appropriate for helping other people with similar problems as yours? | 0 | 1 | 2 | 3 | 8 |
|  |  |  |  |  |  |  |
| AP12 | Do you think the mental health program addresses problems that are common in your community? | 0 | 1 | 2 | 3 | 8 |
|  |  |  |  |  |  |  |
| AP13 | Do you think the skills taught in the mental health program would be relevant to other people like yourself? | 0 | 1 | 2 | 3 | 8 |

|  | | | | | | | |
| --- | --- | --- | --- | --- | --- | --- | --- |
| **2.4 Feasibility** | | | | | | | |
|  | | 0  Not at all | 1  A little bit | 2  A moderate amount | 3  A lot | 8  Don’t know | 9  N/A |
| FS01 | Do you think you would have the necessary time to attend 8-12 weekly sessions of the mental health program? | 0 | 1 | 2 | 3 | 8 | 9 |
| FS02 | Do you think the sessions of the mental health program would be scheduled with enough flexibility to meet your needs? | 0 | 1 | 2 | 3 | 8 | 9 |
| FS03 | Do you think the counselor would be on time when you would come to sessions? | 0 | 1 | 2 | 3 | 8 | 9 |
|  |  |  |  |  |  |  |  |
| FS04 | How possible would it be to get away from your duties (eg. Work, parenting) to attend the mental health program? | 0 | 1 | 2 | 3 | 8 | 9 |
|  |  |  |  |  |  |  |  |
| FS05 | Do you think the amount of time you would spend each week on homework for the mental health program would be manageable? | 0 | 1 | 2 | 3 | 8 | 9 |
|  |  |  |  |  |  |  |  |
| FS06 | Do you think you would have enough money to pay for transport to regularly attend the mental health program? | 0 | 1 | 2 | 3 | 8 | 9 |
|  |  |  |  |  |  |  |  |
| FS07 | Do you think you would have enough money to pay for the other things you would need to regularly attend the mental health program? | 0 | 1 | 2 | 3 | 8 | 9 |
|  |  |  |  |  |  |  |  |
| FS08 | How much would receiving the mental health service described affect your income? | 0 | 1 | 2 | 3 | 8 | 9 |
|  |  |  |  |  |  |  |  |
| FS09 | Do you think you would have the resources (phone, talk time) to communicate with your counselor from | 0 | 1 | 2 | 3 | 8 | 9 |

|  | the mental health program when needed? |  |  |  |  |  |  |
| --- | --- | --- | --- | --- | --- | --- | --- |
|  |  |  |  |  |  |  |  |
| FS10 | Do you think you would have the emotional support you needed from your family and friends to regularly attend the mental health program? | 0 | 1 | 2 | 3 | 8 | 9 |
|  |  |  |  |  |  |  |  |
| FS11 | How much would childcare responsibilities affect your ability to attend the mental health program? | 0 | 1 | 2 | 3 | 8 | 9 |
|  |  |  |  |  |  |  |  |
| FS12 | In general, how safe would you feel to travel to weekly sessions for the mental health program? | 0 | 1 | 2 | 3 | 8 | 9 |
|  |  |  |  |  |  |  |  |
| FS13 | How safe is the place where you would meet your counselor? | 0 | 1 | 2 | 3 | 8 | 9 |
|  |  |  |  |  |  |  |  |
| FS14 | Do you think the place where you would meet your counselor would be confidential? | 0 | 1 | 2 | 3 | 8 | 9 |
|  |  |  |  |  |  |  |  |
| FS15 | Do you believe people in your community could seek help for mental health problems from the program without fear of how others would view them? | 0 | 1 | 2 | 3 | 8 | 9 |

|  | | | | | | |
| --- | --- | --- | --- | --- | --- | --- |
| **2.5 Reach/Access** | | | | | | |
|  | | 0  Not at all | 1  A little bit | 2  A moderate amount | 3  A lot | 8  Don’t know |
| RA01 | Would people in the community be aware that the mental health program is available? | 0 | 1 | 2 | 3 | 8 |
| RA02 | If you sought help, how much of a problem would you have with the amount of time you had to wait to begin the program? | 0 | 1 | 2 | 3 | 8 |
| RA03 | Would most people in the community who need mental health services seek | 0 | 1 | 2 | 3 | 8 |

|  | out the services provided by the program? |  |  |  |  |  |
| --- | --- | --- | --- | --- | --- | --- |
|  |  |  |  |  |  |  |
| RA04 | Would the poorest people in the community who need mental heath services seek out the services provided by the program? | 0 | 1 | 2 | 3 | 8 |
|  |  |  | | | | |
| RA04a | (If not at all or a little bit) What are reasons the poorest people would not seek services? |  | | | | |
|  |  |  |  |  |  |  |
| RA05 | Would women who need mental health services seek out the services provided by the program? | 0 | 1 | 2 | 3 | 8 |
|  |  |  | | | | |
| RA05a | (If not at all or a little bit) What are reasons women would not seek services? |  | | | | |
|  |  |  |  |  |  |  |
| RA06 | Would men who need mental heath services seek out the services provided by the program? | 0 | 1 | 2 | 3 | 8 |
|  |  |  | | | | |
| RA06a | (If not at all or a little bit) What are reasons men would not seek services? |  | | | | |
|  |  |  |  |  |  |  |
| RA07 | Would parents or other caretakers seek the mental health services provided by the program if their children needed it? | 0 | 1 | 2 | 3 | 8 |
|  |  |  | | | | |
| RA07a | (If not at all or a little bit) What are reasons parents or caretakers would not seek services? |  | | | | |
|  |  |  |  |  |  |  |
| RA08 | Would children without parents seek out the services provided by the program? | 0 | 1 | 2 | 3 | 8 |
|  |  |  | | | | |
| RA08a | (If not at all or a little bit) What are reasons children without parents would not seek services? |  | | | | |
|  |  |  | | | | |
| RA09 | Who in your community would not seek the mental health services provided by the program? |  | | | | |

***To be asked after full interview by interviewer**

An **Adoptable** program is a program that people are willing to try and then continue to participate in after they started.

*How much do you think the program described in the story meets this definition of adoptable?*

*Please describe your reasoning. What is wrong or missing from the definition?*

Probes:

1. Are there aspects of the program that should be changed to make it more likely to be adopted? If so, what are they?
2. What else would we need to know about the program to help with understanding how likely it is to be adopted?

| Not at all 1 | A little bit 2 | A moderate amount  3 | A lot 4 |
| --- | --- | --- | --- |

|  | | | | | |
| --- | --- | --- | --- | --- | --- |
| An **Acceptable** program is satisfying and agreeable.  *How much do you think the program described in the story meets this definition of acceptable?* | | | | | |
|  | Not at all 1 | A little bit 2 | A moderate amount  3 | A lot 4 |  |

An **Appropriate** program means that the program fits your needs. It would be an effective way to help your problems and suitable/compatible to your lifestyle and culture.

*How much do you think the program described in the story meets this definition of appropriate?*

*Please describe your reasoning. What is wrong or missing from the definition?*

Probes:

1. Are there aspects of the program that should be changed to make it more appropriate? If so, what are they?
2. What else would we need to know about the program to help with understanding how appropriate it is?

*Please describe your reasoning. What is wrong or missing from the definition?*

Probes:

1. Are there aspects of the program that should be changed to make it more acceptable? If so, what are they?
2. What else would we need to know about the program to help with understanding how acceptable it is?

| Not at all 1 | A little bit 2 | A moderate amount  3 | A lot 4 |
| --- | --- | --- | --- |

A **Feasible** program is a program that you can participate in given your available resources (time, money, family/job obligations, etc.).

*Do you think the program described in the story meets this definition of feasible?*

*Please describe your reasoning. What is wrong or missing from the definition?*

Probes:

1. Are there aspects of the program that should be changed to make it more feasible? If so, what are they?
2. What else would we need to know about the program to help with understanding how feasible it is?

An **Accessible** program is one that would be easily available to people who need the services.

*Do you think the program described in the story meets this definition of accessible?*

*Please describe your reasoning. What is wrong or missing from the definition?*

Probes:

| Not at all 1 | A little bit 2 | A moderate amount  3 | A lot 4 |
| --- | --- | --- | --- |

| Not at all 1 | A little bit 2 | A moderate amount  3 | A lot 4 |
| --- | --- | --- | --- |

1. Are there aspects of the program that should be changed to make it more accessible? If so, what are they?
2. What else would we need to know about the program to help with understanding how accessible it is?
