# Supplementary material for: Measuring implementation in global mental health: validation of a pragmatic implementation science measure in eastern Ukraine using an experimental vignette design
Source: BMC Health Serv Res. 2019 Apr 29;19:262. doi: 10.1186/s12913-019-4097-y (PMC6489318; doi:10.1186/s12913-019-4097-y)
Supplement: Supplementary file 4 — Consumer_Psychiatric Vignette. Vignette depicting current standard of care in Ukraine. (DOCX 13 kb) [file 12913_2019_4097_MOESM4_ESM.docx]

**The description of the existing assistance model in the sphere of mental health in Ukraine.**

The help in the sphere of mental health in Ukraine appears psychiatrists, experts with medical education. In this description it will be a question of assistance to immigrants and veterans of anti-terrorist operation who have the psychological problems caused by a trauma and who are so serious that they considerably limit ability of the person to function and carry out habitual social duties.

A person in need of assistance should apply to the mental hospital at the place of residence or to the neurologist in the district hospital. Psychiatrists are trained as medical doctors and may or may not have experience working with IDPS and veterans and treating trauma. Consultation with a psychiatrist going on the record. To do this, the patient is issued a personal card, which entered the basic data in the registry: age, address, passport details. The patient is assigned to the consultation from a psychiatrist, who conducts the primary diagnosis and he makes a diagnosis if necessary. The meeting lasts about 20-30 minutes. Generally, treatment is given in the case of pronounced problems with sleep, anxiety and panic attacks. If the main problem is the patient's complaints of sleep, he was appointed medical treatment sleeping pills. The patient is asked to take sleeping pills 1 time per day during the week. If the main problem is the state of anxiety or fear, he appointed daily tranquilizers, to be taken 7-14 days twice a day. If the patient has depression, for example, he feels a strong depression, lack of appetite, loss of interest and as it is assigned antidepressant treatment, which lasts 6 weeks. The same treatment is given in the case of strong panic attacks that do not allow it, for example, go out. Preparations need to be taken 1 times a day. If the patient has expressed an alcohol problem, your doctor may send it to the Drug Dispensary. As a rule, the doctor recommends to the patient to find the psychologist. He can recommend the psychologist practicing privately or not to recommend anybody specifically. If the symptoms disappear, repeat meeting is not appointed. If there is no improvement or worsening of symptoms, the patient can go to a psychiatrist again, as a rule, on its own initiative, and to come to the re-consultation to amend the scheme of taking the drugs.

The psychiatrist can send the patient to a hospital of borderlines for passing of two-week hospitalization. It occurs if symptoms don't disappear or amplify. For example, the patient can endure panic attacks of such force that isn't able to go outside or work in collective. In this case the diagnosis – neurosis is made to him. Further treatment, as a rule is medical, in some hospitals the group psychotherapy is possible.

Consent to drug out-patient treatment or treatment in a hospital is only voluntary. As a rule, the patient is registered and brought in the psychiatric database. It can lead to certain restrictions. For example, if you want to get a driver's license, he will be assigned to the medical commission. At the request of the patient, he/she cannot be registered and treatment of a psychiatrist does not have any negative social or legal consequences.
