# Supplementary material for: Measuring implementation in global mental health: validation of a pragmatic implementation science measure in eastern Ukraine using an experimental vignette design
Source: BMC Health Serv Res. 2019 Apr 29;19:262. doi: 10.1186/s12913-019-4097-y (PMC6489318; doi:10.1186/s12913-019-4097-y)
Supplement: Supplementary file 5 — UkraineConsumerAssessmentTool_ FieldReady _ENG. Full quantitative assessment used in the study. (DOCX 114 kb) [file 12913_2019_4097_MOESM5_ESM.docx]

**Form: AMHRG_UkraineConsumerAssessmentTool**

320 Questions

====================================================

**1.Enter the client ID number.**

**ID**

**2.Please double check the client ID number and re-enter it.**

**3.Enter your interviewer ID number.**

**ID**

**4.Enter today's date.**

**5.Which interview is this?**

Choose one response

- First interview

- Second interview

**6.Which site is this?**

Choose one response

- Kiev

- Zaporizhia

**7.Which Vignette?**

Choose one response

- Ukraine Psychiatric

- CETA

**8. Thank you for agreeing to participate in the survey. Below are four practice questions to help you get used to taking the survey on the tablet. After you have tried the practice questions, please let the interviewer know if you have any concerns or questions.**

**9.Practice Question 1: Please enter how many days there are in a week.**

**10.Practice Question 2: Check the box next to the sports that you like. If you don't like any of the sports listed, check the box that says "Other".**

Choose all that apply

- Soccer

- Aerobics

- Weight training

- Tennis

- Skiing

- Ice hockey

- Volleyball

- Gymnastics

- Basketball

- Skateboarding

- Rugby

- Yoga

- Other

**11.Practice Question 3: Is Ukraine a country in Europe?**

Choose one response

- Yes

- No

**12.Practice Question 4: How often do you talk on the phone?**

Choose one response

- None of the time

- A little of the time

- Most of the time

- About all of the time

**13. The next section asks about demographic information.**

**14.Sex**

Choose one response

- Male

- Female

**15.Age in years**

**16.Current marital status:**

Choose one response

- Single

- Married

- Widowed

- Divorced

**17.What is the highest level of education you have completed?**

Choose one response

- None

- Primary (1-8)

- High school (9-12)

- University (13-16)

- More than university

**18. The next questions ask about a number of difficult or stressful things that sometimes happen to people. For each event check one or more of the boxes to the right to indicate that:**

**(a) it happened to you personally; (b) you witnessed it happen to someone else; (c) you learned about it happening to a close family member or close friend, or (d) it doesn't apply to you.**

**Be sure to consider your entire life (growing up as well as adulthood) as you go through the list of events.**

**19.Natural disaster (for example: flood, cyclone, earthquake)**

Choose all that apply

- Happened to me

- Witnessed it

- Learned about it

- Not applicable

- No response

**20.Fire or explosion**

Choose all that apply

- Happened to me

- Witnessed it

- Learned about it

- Not applicable

- No response

**21.Transportation accident (for example: car accident, boat accident, train wreck, plane crash)**

Choose all that apply

- Happened to me

- Witnessed it

- Learned about it

- Doesn't apply

- No response

**22.Serious accident at work, home, or during recreational activity**

Choose all that apply

- Happened to me

- Witnessed it

- Learned about it

- Doesn't apply

- No response

**23.Exposure to toxic substance (for example: dangerous chemicals, radiation)**

Choose all that apply

- Happened to me

- Witnessed it

- Learned about it

- Doesn't apply

- No response

**24.Physical assault (for example: being attacked, hit, slapped, kicked, beaten up)**

Choose all that apply

- Happened to me

- Witnessed it

- Learned about it

- Doesn't apply

- No response

**25.Assault with a weapon (for example: being shot, stabbed, threatened with a knife, gun, bomb)**

Choose all that apply

- Happened to me

- Witnessed it

- Learned about it

- Doesn't apply

- No response

**26.Sexual assault (rape, attempted rape, made to performa any type of sexual act through force or threat)**

Choose all that apply

- Happened to me

- Witnessed it

- Learned about it

- Doesn't apply

- No response

**27.Other unwanted or uncomfortable sexual experience**

Choose all that apply

- Happened to me

- Witnessed it

- Learned about it

- Doesn't apply

- No response

**28.Combat or exposure to a war-zone (in the military or as a civilian)**

Choose all that apply

- Happened to me

- Witnessed it

- Learned about it

- Doesn't apply

- No response

**29.Captivity (for example: being kidnapped, abducted, held hostage, prisoner of war)**

Choose all that apply

- Happened to me

- Witnessed it

- Learned about it

- Doesn't apply

- No response

**30.Life-threatening illness or injury**

Choose all that apply

- Happened to me

- Witnessed it

- Learned about it

- Doesn't apply

- No response

**31.Severe human suffering**

Choose all that apply

- Happened to me

- Witnessed it

- Learned about it

- Doesn't apply

- No response

**32.Sudden, violent death (for example: homicide, suicide)**

Choose all that apply

- Happened to me

- Witnessed it

- Learned about it

- Doesn't apply

- No response

**33.Sudden accidental death**

Choose all that apply

- Happened to me

- Witnessed it

- Learned about it

- Doesn't apply

- No response

**34.Serious injury, harm, or death you caused to someone else**

Choose all that apply

- Happened to me

- Witnessed it

- Learned about it

- Doesn't apply

- No response

**35.Forced displacement from home**

Choose all that apply

- Happened to me

- Witnessed it

- Learned about it

- Doesn't apply

- No response

**36.Lost contact with loved ones and fear for their safety**

Choose all that apply

- Happened to me

- Witnessed it

- Learned about it

- Doesn't apply

- No response

**37.Starvation or fear of starvation**

Choose all that apply

- Happened to me

- Witnessed it

- Learned about it

- Doesn't apply

- No response

**38.Sudden loss of possessions to the point of poverty and concern about how one will survive**

Choose all that apply

- Happened to me

- Witnessed it

- Learned about it

- Doesn't apply

- No response

**39.Any other very stressful event or experience**

Choose all that apply

- Happened to me

- Witnessed it

- Learned about it

- Doesn't apply

- No response

**40. The following are symptoms that people sometimes have after experiencing hurtful or terrifying events in their lives. Please read each one carefully and decide how much of the time the symptoms bothered you in the last two weeks.**

**41.Feeling afraid/fearful**

Choose one response

- None of the time

- A little of the time

- Most of the time

- Almost all of the time

- No response

**42.Feeling isolated**

Choose one response

- None of the time

- A little of the time

- Most of the time

- Almost all of the time

- No response

**43.Feeling detached from others**

Choose one response

- None of the time

- A little of the time

- Most of the time

- Almost all of the time

- No response

**44.Unable to experience positive emotions**

Choose one response

- None of the time

- A little of the time

- Most of the time

- Almost all of the time

- No response

**45.Repeated or disturbing memories, thoughts, or images about the trauma**

Choose one response

- None of the time

- A little of the time

- Most of the time

- Almost all of the time

- No response

**46.Repeated trauma-related dreams/nightmares**

Choose one response

- None of the time

- A little of the time

- Most of the time

- Almost all of the time

- No response

**47.Feeling a sense of loss**

Choose one response

- None of the time

- A little of the time

- Most of the time

- Almost all of the time

- No response

**48.Intimate relationship problems**

Choose one response

- None of the time

- A little of the time

- Most of the time

- Almost all of the time

- No response

**49.Reckless or self-destructive behavior**

Choose one response

- None of the time

- A little of the time

- Most of the time

- Almost all of the time

- No response

**50.Feeling agitated**

Choose one response

- None of the time

- A little of the time

- Most of the time

- Almost all of the time

- No response

**51.Feeling emotionally upset when something reminded you of the stressful event**

Choose one response

- None of the time

- A little of the time

- Most of the time

- Almost all of the time

- No response

**52.Feeling that no one understands**

Choose one response

- None of the time

- A little of the time

- Most of the time

- Almost all of the time

- No response

**53.Fights or difficulty getting along with family**

Choose one response

- None of the time

- A little of the time

- Most of the time

- Almost all of the time

- No response

**54.Feeling unable to cope**

Choose one response

- None of the time

- A little of the time

- Most of the time

- Almost all of the time

- No response

**55.Angry outbursts**

Choose one response

- None of the time

- A little of the time

- Most of the time

- Almost all of the time

- No response

**56.Feeling jumpy or easily startled**

Choose one response

- None of the time

- A little of the time

- Most of the time

- Almost all of the time

- No response

**57.Feeling guilty**

Choose one response

- None of the time

- A little of the time

- Most of the time

- Almost all of the time

- No response

**58.Unable to socialize with others**

Choose one response

- None of the time

- A little of the time

- Most of the time

- Almost all of the time

- No response

**59.Feeling super alert, watchful, or on guard**

Choose one response

- None of the time

- A little of the time

- Most of the time

- Almost all of the time

- No response

**60.Acting or feeling as if the past stressful event is happening again, as if you were reliving it**

Choose one response

- None of the time

- A little of the time

- Most of the time

- Almost all of the time

- No response

**61.A physical reaction when reminded of the past stressful event. For example: heart racing, sweating, shaking, rapid breathy, feeling faint or nauseous.**

Choose one response

- None of the time

- A little of the time

- Most of the time

- Almost all of the time

- No response

**62.Avoiding thoughts/memories related to the past stressful event**

Choose one response

- None of the time

- A little of the time

- Most of the time

- Almost all of the time

- No response

**63.Avoiding activities or situations that remind me of the past stressful event**

Choose one response

- None of the time

- A little of the time

- Most of the time

- Almost all of the time

- No response

**64.Unable to remember parts of the past stressful event**

Choose one response

- None of the time

- A little of the time

- Most of the time

- Almost all of the time

- No response

**65.Blaming yourself or others for what happened**

Choose one response

- None of the time

- A little of the time

- Most of the time

- Almost all of the time

- No response

**66.Feeling a loss of your sense of self**

Choose one response

- None of the time

- A little of the time

- Most of the time

- Almost all of the time

- No response

**67.Feeling that I have no one to rely on**

Choose one response

- None of the time

- A little of the time

- Most of the time

- Almost all of the time

- No response

**68.Feeling a loss of faith**

Choose one response

- None of the time

- A little of the time

- Most of the time

- Almost all of the time

- No response

**69.Feeling that your surroundings or the environment around me are not real**

Choose one response

- None of the time

- A little of the time

- Most of the time

- Almost all of the time

- No response

**70.Frequently trembling or shaking**

Choose one response

- None of the time

- A little of the time

- Most of the time

- Almost all of the time

- No response

**71.Feeling apathy**

Choose one response

- None of the time

- A little of the time

- Most of the time

- Almost all of the time

- No response

**72.Not being able to remember things or being forgetful**

Choose one response

- None of the time

- A little of the time

- Most of the time

- Almost all of the time

- No response

**73. I would like to ask you questions about how things have been for you in the last two weeks. When you answer each question, I would like you to think back just over the last two weeks.**

**In the past two weeks how often has each of the following problems occurred…**

**74.Feeling sad**

Choose one response

- None of the time

- A little of the time

- Most of the time

- Almost all of the time

- No response

**75.Feeling no interest in things/less interest in daily activities**

Choose one response

- None of the time

- A little of the time

- Most of the time

- Almost all of the time

- No response

**76.Crying easily**

Choose one response

- None of the time

- A little of the time

- Most of the time

- Almost all of the time

- No response

**77.Feeling hopless about the future**

Choose one response

- None of the time

- A little of the time

- Most of the time

- Almost all of the time

- No response

**78.Feeling lonely or socially withdrawn; feeling isolated**

Choose one response

- None of the time

- A little of the time

- Most of the time

- Almost all of the time

- No response

**79.Avoiding others**

Choose one response

- None of the time

- A little of the time

- Most of the time

- Almost all of the time

- No response

**80.Feeling tired, low in energy, or slowed down**

Choose one response

- None of the time

- A little of the time

- Most of the time

- Almost all of the time

- No response

**81.Weighing too little**

Choose one response

- None of the time

- A little of the time

- Most of the time

- Almost all of the time

- No response

**82.Weighing too much**

Choose one response

- None of the time

- A little of the time

- Most of the time

- Almost all of the time

- No response

**83.Problems with my appetite**

Choose one response

- None of the time

- A little of the time

- Most of the time

- Almost all of the time

- No response

**84.Problems with your sleep; Disturbed sleep**

Choose one response

- None of the time

- A little of the time

- Most of the time

- Almost all of the time

- No response

**85.Feeling of being trapped or caught**

Choose one response

- None of the time

- A little of the time

- Most of the time

- Almost all of the time

- No response

**86.Worrying too much about things**

Choose one response

- None of the time

- A little of the time

- Most of the time

- Almost all of the time

- No response

**87.Feelings of worthlessness**

Choose one response

- None of the time

- A little of the time

- Most of the time

- Almost all of the time

- No response

**88.Headache**

Choose one response

- None of the time

- A little of the time

- Most of the time

- Almost all of the time

- No response

**89.Stomach aches**

Choose one response

- None of the time

- A little of the time

- Most of the time

- Almost all of the time

- No response

**90.Other bodily aches and pains**

Choose one response

- None of the time

- A little of the time

- Most of the time

- Almost all of the time

- No response

**91.Feeling angry**

Choose one response

- None of the time

- A little of the time

- Most of the time

- Almost all of the time

- No response

**92.Thinking too much**

Choose one response

- None of the time

- A little of the time

- Most of the time

- Almost all of the time

- No response

**93.Feeling confused**

Choose one response

- None of the time

- A little of the time

- Most of the time

- Almost all of the time

- No response

**94.Feeling weakness in your heart**

Choose one response

- None of the time

- A little of the time

- Most of the time

- Almost all of the time

- No response

**95.Heart palpitations**

Choose one response

- None of the time

- A little of the time

- Most of the time

- Almost all of the time

- No response

**96.Feeling as though your heart or chest is heavy**

Choose one response

- None of the time

- A little of the time

- Most of the time

- Almost all of the time

- No response

**97.Feeling pressure on your heart or chest**

Choose one response

- None of the time

- A little of the time

- Most of the time

- Almost all of the time

- No response

**98.Pain in your heart or chest**

Choose one response

- None of the time

- A little of the time

- Most of the time

- Almost all of the time

- No response

**99.Moving or speaking so slowly or fast that others have noticed**

Choose one response

- None of the time

- A little of the time

- Most of the time

- Almost all of the time

- No response

**100.Difficulty concentrating**

Choose one response

- None of the time

- A little of the time

- Most of the time

- Almost all of the time

- No response

**101.Difficulty doing your usual activities at home or work**

Choose one response

- None of the time

- A little of the time

- Most of the time

- Almost all of the time

- No response

**102.Thoughts of wanting to kill yourself; propensity for suicide**

Choose one response

- None of the time

- A little of the time

- Most of the time

- Almost all of the time

- No response

**103. I would like to ask you questions about how things have been for you in the last two weeks. When you answer each question, I would like you to think back just over the last two weeks.**

**In the past two weeks how often has each of the following problems occurred…**

**104.Feeling misunderstood by others in the community**

Choose one response

- None of the time

- A little of the time

- Most of the time

- Almost all of the time

- No response

**105.Feeling helpless**

Choose one response

- None of the time

- A little of the time

- Most of the time

- Almost all of the time

- No response

**106.Humiliation of human dignity**

Choose one response

- None of the time

- A little of the time

- Most of the time

- Almost all of the time

- No response

**107.Feeling inadequate**

Choose one response

- None of the time

- A little of the time

- Most of the time

- Almost all of the time

- No response

**108.You are unable to influence anything**

Choose one response

- None of the time

- A little of the time

- Most of the time

- Almost all of the time

- No response

**109.Not feeling accepted by my family**

Choose one response

- None of the time

- A little of the time

- Most of the time

- Almost all of the time

- No response

**110.Not feeling accepted by the community**

Choose one response

- None of the time

- A little of the time

- Most of the time

- Almost all of the time

- No response

**111.Having difficulties adapting to the community**

Choose one response

- None of the time

- A little of the time

- Most of the time

- Almost all of the time

- No response

**112.Feeling mistrustful/suspicious**

Choose one response

- None of the time

- A little of the time

- Most of the time

- Almost all of the time

- No response

**113.Feeling betrayed by the community**

Choose one response

- None of the time

- A little of the time

- Most of the time

- Almost all of the time

- No response

**114.Feeling betrayed by the government**

Choose one response

- None of the time

- A little of the time

- Most of the time

- Almost all of the time

- No response

**115.Feeling of loss**

Choose one response

- None of the time

- A little of the time

- Most of the time

- Almost all of the time

- No response

**116.Longing**

Choose one response

- None of the time

- A little of the time

- Most of the time

- Almost all of the time

- No response

**117.Feeling abandoned**

Choose one response

- None of the time

- A little of the time

- Most of the time

- Almost all of the time

- No response

**118.Lack of emotional stability**

Choose one response

- None of the time

- A little of the time

- Most of the time

- Almost all of the time

- No response

**119.Feeling aggressive towards others**

Choose one response

- None of the time

- A little of the time

- Most of the time

- Almost all of the time

- No response

**120.Acting verbally aggressively towards others (e.g. verbal altercations; verbally assaulting others)**

Choose one response

- None of the time

- A little of the time

- Most of the time

- Almost all of the time

- No response

**121.Acting physically aggressive towards others (e.g. physical altercations; assaulting others)**

Choose one response

- None of the time

- A little of the time

- Most of the time

- Almost all of the time

- No response

**122.Feeling closed up**

Choose one response

- None of the time

- A little of the time

- Most of the time

- Almost all of the time

- No response

**123.Experiencing excitement**

Choose one response

- None of the time

- A little of the time

- Most of the time

- Almost all of the time

- No response

**124.Psyche is disturbed**

Choose one response

- None of the time

- A little of the time

- Most of the time

- Almost all of the time

- No response

**125.Feeling irritated or irritable**

Choose one response

- None of the time

- A little of the time

- Most of the time

- Almost all of the time

- No response

**126.Feeling of shock**

Choose one response

- None of the time

- A little of the time

- Most of the time

- Almost all of the time

- No response

**127.Feeling misunderstood by my family/ friends**

Choose one response

- None of the time

- A little of the time

- Most of the time

- Almost all of the time

- No response

**128.Dwelling on your problems**

Choose one response

- None of the time

- A little of the time

- Most of the time

- Almost all of the time

- No response

**129. I would like to ask you questions about how things have been for you in the last two weeks. When you answer each question, I would like you to think back just over the last two weeks.**

**In the past two weeks how often has each of the following problems occurred…**

**130.Suddenly scared for no reason**

Choose one response

- None of the time

- A little of the time

- Most of the time

- Almost all of the time

- No response

**131.Feeling fearful**

Choose one response

- None of the time

- A little of the time

- Most of the time

- Almost all of the time

- No response

**132.Faintness, dizziness, or weakness**

Choose one response

- None of the time

- A little of the time

- Most of the time

- Almost all of the time

- No response

**133.Nervousness or shakiness inside**

Choose one response

- None of the time

- A little of the time

- Most of the time

- Almost all of the time

- No response

**134.Heart pounding or racing**

Choose one response

- None of the time

- A little of the time

- Most of the time

- Almost all of the time

- No response

**135.Trembling**

Choose one response

- None of the time

- A little of the time

- Most of the time

- Almost all of the time

- No response

**136.Feeling tense or keyed up**

Choose one response

- None of the time

- A little of the time

- Most of the time

- Almost all of the time

- No response

**137.Spells of terror or panic**

Choose one response

- None of the time

- A little of the time

- Most of the time

- Almost all of the time

- No response

**138.Feeling restless, can't sit still**

Choose one response

- None of the time

- A little of the time

- Most of the time

- Almost all of the time

- No response

**139. The next section asks about alcohol, tobacco products and other drugs. The questions are about your experience of using these substances across your lifetime and in the past three months. These substances can be smoked, swallowed, snorted, inhaled, injected, or taken in the form of pills (show drug card).**

**Some of the substances listed may be prescribed by a doctor (like amphetamines, sedatives, pain medications). For this interview, do not record medications that are used as prescribed by your doctor. However, if you have taken such medications for reasons other than prescription, or taken them more frequently or at higher doses than prescribed, please indicate this is so. While we are also interested in knowing about your use of various illicit drugs, please be assured that information on such use will be treated as strictly confidential.**

**140.In your life, have you ever used tobacco products (cigarettes, chewing tobacco, cigars, etc.)?**

Choose one response

- no

- yes

- no response

**141.In the past three months, how often have you used tobacco products?**

Choose one response

- Never

- Once or Twice

- Monthly

- Weekly

- Daily or Almost Daily

- No response

**142.During the past three months, how often have you had a strong desire to use tobacco products?**

Choose one response

- Never

- Once or Twice

- Monthly

- Weekly

- Daily or Almost Daily

- No response

**143.During the past three months, how often has your use of tobacco products led to health, social, legal, or financial problems?**

Choose one response

- Never

- Once or Twice

- Monthly

- Weekly

- Daily or Almost Daily

- No response

**144.During the past three months, how often have you failed to do what was normally expected of you because of your use of tobacco products?**

Choose one response

- Never

- Once or Twice

- Monthly

- Weekly

- Daily or Almost Daily

- No response

**145.Has a friend or relative or anyone else ever expressed concern about your use of tobacco products?**

Choose one response

- No, Never

- Yes, in the past 3 months

- Yes, but not in the past 3 months

- No response

**146.Have you ever tried and failed to control, cut down, or stop using tobacco products?**

Choose one response

- No, Never

- Yes, in the past 3 months

- Yes, but not in the past 3 months

- No response

**147.In your life, have you ever used alcoholic beverages (beer, wine, spirits, etc.)?**

Choose one response

- no

- yes

- no response

**148.During the past three months, how often have you used alcoholic beverages?**

Choose one response

- Never

- Once or Twice

- Monthly

- Weekly

- Daily or Almost Daily

- No response

**149.During the past three months, how often have you had a strong desire to use alcoholic beverages?**

Choose one response

- Never

- Once or Twice

- Monthly

- Weekly

- Daily or Almost Daily

- No response

**150.During the past three months, how often has your use of alcoholic beverages led to health, social, legal, or financial problems?**

Choose one response

- Never

- Once or Twice

- Monthly

- Weekly

- Daily or Almost Daily

- No response

**151.During the past three months, how often have you failed to do what was normally expected of you because of your use of alcoholic beverages?**

Choose one response

- Never

- Once or Twice

- Monthly

- Weekly

- Daily or Almost Daily

- No response

**152.Has a friend or relative or anyone else ever expressed concern about your use of alcoholic beverages?**

Choose one response

- No, Never

- Yes, in the past 3 months

- Yes, but not in the past 3 months

- No response

**153.Have you ever tried and failed to control, cut down, or stop using alcoholic beverages?**

Choose one response

- No, Never

- Yes, in the past 3 months

- Yes, but not in the past 3 months

- No response

**154.In your life, have you ever used cannabis (marijuna, pot, grass, hash, etc.)?**

Choose one response

- no

- yes

- no response

**155.During the past three months, how often have you used cannabis?**

Choose one response

- Never

- Once or Twice

- Monthly

- Weekly

- Daily or Almost Daily

- No response

**156.During the past three months, how often have you had a strong desire to use cannabis?**

Choose one response

- Never

- Once or Twice

- Monthly

- Weekly

- Daily or Almost Daily

- No response

**157.During the past three months, how often has your use of cannabis led to health, social, legal, or financial problems?**

Choose one response

- Never

- Once or Twice

- Monthly

- Weekly

- Daily or Almost Daily

- No response

**158.During the past three months, how often have you failed to do what was normally expected of you because of your use of cannabis?**

Choose one response

- Never

- Once or Twice

- Monthly

- Weekly

- Daily or Almost Daily

- No response

**159.Has a friend or relative or anyone else ever expressed concern about your use of cannabis?**

Choose one response

- No, Never

- Yes, in the past 3 months

- Yes, but not in the past 3 months

- No response

**160.Have you ever tried and failed to control, cut down, or stop using cannabis?**

Choose one response

- No, Never

- Yes, in the past 3 months

- Yes, but not in the past 3 months

- No response

**161.In your life, have you ever used cocaine (coke, crack, etc.)?**

Choose one response

- no

- yes

- no response

**162.During the past three months, how often have you used cocaine?**

Choose one response

- Never

- Once or Twice

- Monthly

- Weekly

- Daily or Almost Daily

- No response

**163.During the past three months, how often have you had a strong desire to use cocaine?**

Choose one response

- Never

- Once or Twice

- Monthly

- Weekly

- Daily or Almost Daily

- No response

**164.During the past three months, how often has your use of cocaine led to health, social, legal, or financial problems?**

Choose one response

- Never

- Once or Twice

- Monthly

- Weekly

- Daily or Almost Daily

- No response

**165.During the past three months, how often have you failed to do what was normally expected of you because of your use of cocaine?**

Choose one response

- Never

- Once or Twice

- Monthly

- Weekly

- Daily or Almost Daily

- No response

**166.Has a friend or relative or anyone else ever expressed concern about your use of cocaine?**

Choose one response

- No, Never

- Yes, in the past 3 months

- Yes, but not in the past 3 months

- No response

**167.Have you ever tried and failed to control, cut down, or stop using cocaine?**

Choose one response

- No, Never

- Yes, in the past 3 months

- Yes, but not in the past 3 months

- No response

**168.In your life, have you ever used amphetamine type stimulants (speed, diet pills, ecstasy, etc.)?**

Choose one response

- no

- yes

- no response

**169.During the past three months, how often have you used amphetamine type stimulants?**

Choose one response

- Never

- Once or Twice

- Monthly

- Weekly

- Daily or Almost Daily

- No response

**170.During the past three months, how often have you had a strong desire to use amphetamine type stimulants?**

Choose one response

- Never

- Once or Twice

- Monthly

- Weekly

- Daily or Almost Daily

- No response

**171.During the past three months, how often has your use of amphetamine type stimulants led to health, social, legal, or financial problems?**

Choose one response

- Never

- Once or Twice

- Monthly

- Weekly

- Daily or Almost Daily

- No response

**172.During the past three months, how often have you failed to do what was normally expected of you because of your use of amphetamine type stimulants?**

Choose one response

- Never

- Once or Twice

- Monthly

- Weekly

- Daily or Almost Daily

- No response

**173.Has a friend or relative or anyone else ever expressed concern about your use of amphetamine type stimulants?**

Choose one response

- No, Never

- Yes, in the past 3 months

- Yes, but not in the past 3 months

- No response

**174.Have you ever tried and failed to control, cut down, or stop using amphetamine type stimulants?**

Choose one response

- No, Never

- Yes, in the past 3 months

- Yes, but not in the past 3 months

- No response

**175.In your life, have you ever used inhalants (nitrous, glue, petrol, paint thinner, etc.)?**

Choose one response

- no

- yes

- no response

**176.During the past three months, how often have you used inhalants?**

Choose one response

- Never

- Once or Twice

- Monthly

- Weekly

- Daily or Almost Daily

- No response

**177.During the past three months, how often have you had a strong desire to use inhalants?**

Choose one response

- Never

- Once or Twice

- Monthly

- Weekly

- Daily or Almost Daily

- No response

**178.During the past three months, how often has your use of inhalants led to health, social, legal, or financial problems?**

Choose one response

- Never

- Once or Twice

- Monthly

- Weekly

- Daily or Almost Daily

- No response

**179.During the past three months, how often have you failed to do what was normally expected of you because of your use of inhalants?**

Choose one response

- Never

- Once or Twice

- Monthly

- Weekly

- Daily or Almost Daily

- No response

**180.Has a friend or relative or anyone else ever expressed concern about your use of inhalants?**

Choose one response

- No, Never

- Yes, in the past 3 months

- Yes, but not in the past 3 months

- No response

**181.Have you ever tried and failed to control, cut down, or stop using inhalants?**

Choose one response

- No, Never

- Yes, in the past 3 months

- Yes, but not in the past 3 months

- No response

**182.In your life, have you ever used sedatives or sleeping pills (Valium, Serepax, Rohypnol, etc.)?**

Choose one response

- no

- yes

- no response

**183.During the past three months, how often have you used sedatives or sleeping pills?**

Choose one response

- Never

- Once or Twice

- Monthly

- Weekly

- Daily or Almost Daily

- No response

**184.During the past three months, how often have you had a strong desire to use sedatives or sleeping pills?**

Choose one response

- Never

- Once or Twice

- Monthly

- Weekly

- Daily or Almost Daily

- No response

**185.During the past three months, how often has your use of sedatives or sleeping pills led to health, social, legal, or financial problems?**

Choose one response

- Never

- Once or Twice

- Monthly

- Weekly

- Daily or Almost Daily

- No response

**186.During the past three months, how often have you failed to do what was normally expected of you because of your use of sedatives or sleeping pills?**

Choose one response

- Never

- Once or Twice

- Monthly

- Weekly

- Daily or Almost Daily

- No response

**187.Has a friend or relative or anyone else ever expressed concern about your use of sedatives or sleeping pills?**

Choose one response

- No, Never

- Yes, in the past 3 months

- Yes, but not in the past 3 months

- No response

**188.Have you ever tried and failed to control, cut down, or stop using sedatives or sleeping pills?**

Choose one response

- No, Never

- Yes, in the past 3 months

- Yes, but not in the past 3 months

- No response

**189.In your life, have you ever used hallucinogens (LSD, acid, mushrooms, PCP, Special K, etc.)?**

Choose one response

- no

- yes

- no response

**190.During the past three months, how often have you used hallucinogens?**

Choose one response

- Never

- Once or Twice

- Monthly

- Weekly

- Daily or Almost Daily

- No response

**191.During the past three months, how often have you had a strong desire to use hallucinogens?**

Choose one response

- Never

- Once or Twice

- Monthly

- Weekly

- Daily or Almost Daily

- No response

**192.During the past three months, how often has your use of hallucinogens led to health, social, legal, or financial problems?**

Choose one response

- Never

- Once or Twice

- Monthly

- Weekly

- Daily or Almost Daily

- No response

**193.During the past three months, how often have you failed to do what was normally expected of you because of your use of hallucinogens?**

Choose one response

- Never

- Once or Twice

- Monthly

- Weekly

- Daily or Almost Daily

- No response

**194.Has a friend or relative or anyone else ever expressed concern about your use of hallucinogens?**

Choose one response

- No, Never

- Yes, in the past 3 months

- Yes, but not in the past 3 months

- No response

**195.Have you ever tried and failed to control, cut down, or stop using hallucinogens?**

Choose one response

- No, Never

- Yes, in the past 3 months

- Yes, but not in the past 3 months

- No response

**196.In your life, have you ever used opioids (heroin, morphine, methadone, codeine, etc.)?**

Choose one response

- no

- yes

- no response

**197.During the past three months, how often have you used opioids?**

Choose one response

- Never

- Once or Twice

- Monthly

- Weekly

- Daily or Almost Daily

- No response

**198.During the past three months, how often have you had a strong desire to use opioids?**

Choose one response

- Never

- Once or Twice

- Monthly

- Weekly

- Daily or Almost Daily

- No response

**199.During the past three months, how often has your use of opioids led to health, social, legal, or financial problems?**

Choose one response

- Never

- Once or Twice

- Monthly

- Weekly

- Daily or Almost Daily

- No response

**200.During the past three months, how often have you failed to do what was normally expected of you because of your use of opioids?**

Choose one response

- Never

- Once or Twice

- Monthly

- Weekly

- Daily or Almost Daily

- No response

**201.Has a friend or relative or anyone else ever expressed concern about your use of opioids?**

Choose one response

- No, Never

- Yes, in the past 3 months

- Yes, but not in the past 3 months

- No response

**202.Have you ever tried and failed to control, cut down, or stop using opioids?**

Choose one response

- No, Never

- Yes, in the past 3 months

- Yes, but not in the past 3 months

- No response

**203.In your life, have you ever used any other substance?**

Choose one response

- no

- yes

- no response

**204.Which other drug or substance did you use?**

**205.During the past three months, how often have you used this substance?**

Choose one response

- Never

- Once or Twice

- Monthly

- Weekly

- Daily or Almost Daily

- No response

**206.During the past three months, how often have you had a strong desire to use this substance?**

Choose one response

- Never

- Once or Twice

- Monthly

- Weekly

- Daily or Almost Daily

- No response

**207.During the past three months, how often has your use of this substance led to health, social, legal, or financial problems?**

Choose one response

- Never

- Once or Twice

- Monthly

- Weekly

- Daily or Almost Daily

- No response

**208.During the past three months, how often have you failed to do what was normally expected of you because of your use of this substance?**

Choose one response

- Never

- Once or Twice

- Monthly

- Weekly

- Daily or Almost Daily

- No response

**209.Has a friend or relative or anyone else ever expressed concern about your use of this substance?**

Choose one response

- No, Never

- Yes, in the past 3 months

- Yes, but not in the past 3 months

- No response

**210.Have you ever tried and failed to control, cut down, or stop using this substance?**

Choose one response

- No, Never

- Yes, in the past 3 months

- Yes, but not in the past 3 months

- No response

**211.Have you ever used any drug by injection? (Non-medical use only.)**

Choose one response

- no, never

- yes, in the past 3 months

- yes, but not in the past 3 months

- no response

**212.Was this drug injected more than once per week?**

Choose one response

- no

- yes

- no response

**213.Was this drug injected three or more days in a row?**

Choose one response

- no

- yes

- no response

**214. This section asks about difficulties you have doing daily activities because of the way you are thinking or feeling. Think back over the past 30 days and answer these questions, thinking about how much difficulty you had doing the following activities because of the way you have been thinking or feeling. For each question, please select only one response.**

**In the past 30 days, how much difficulty did you have in...**

**215.Standing for long periods such as 30 minutes?**

Choose one response

- None

- Mild

- Moderate

- Severe

- Extreme or could not do

- No response

**216.Taking care of your household responsibilities?**

Choose one response

- None

- Mild

- Moderate

- Severe

- Extreme or could not do

- No response

**217.Learning a new task (for example: learning how to get to a new place)?**

Choose one response

- None

- Mild

- Moderate

- Severe

- Extreme or could not do

- No response

**218.How much of a problem did you have joining community activities or cultural events (for example festivals, religious or other activities) in the same way as anyone else can?**

Choose one response

- None

- Mild

- Moderate

- Severe

- Extreme or could not do

- No response

**219.How much have you been emotionally affected by your health problems?**

Choose one response

- None

- Mild

- Moderate

- Severe

- Extreme or could not do

- No response

**220.Concentrating on doing something for ten minutes?**

Choose one response

- None

- Mild

- Moderate

- Severe

- Extreme or could not do

- No response

**221.Walking a long distance such as a kilometer [or equivalent]?**

Choose one response

- None

- Mild

- Moderate

- Severe

- Extreme or could not do

- No response

**222.Washing your whole body?**

Choose one response

- None

- Mild

- Moderate

- Severe

- Extreme or could not do

- No response

**223.Getting dressed?**

Choose one response

- None

- Mild

- Moderate

- Severe

- Extreme or could not do

- No response

**224.Dealing with people you do not know?**

Choose one response

- None

- Mild

- Moderate

- Severe

- Extreme or could not do

- No response

**225.Maintaining a friendship?**

Choose one response

- None

- Mild

- Moderate

- Severe

- Extreme or could not do

- No response

**226.Your day-to-day work?**

Choose one response

- None

- Mild

- Moderate

- Severe

- Extreme or could not do

- No response

**227.Overall in the past 30 days, how many days were these difficulties present?**

**228.In the past 30 days, for how many days were you totally unable to carry out your usual activities or work because of any health condition?**

**229.In the past 30 days, not counting the days that you were totally unable, for how many days did you cut back or reduce your usual activities or work because of any health condition?**

**230. Here are some activities and tasks that others in your community have told us are important to them. For each one please respond with how much more difficulty you currently have doing it COMPARED TO OTHER MEN/WOMEN OF YOUR AGE. You should indicate whether you are having one of the following: no more difficulty, a little bit more, a moderate amount more, a lot more, or you often cannot do. Please look at the pictures on the sheet to help you answer the questions. If the task or activity is not relevant to you (for example a question about children and you do not have children), please mark n/a. The reasons why you are having difficulty with the task do not matter and could be different for different tasks, such as lack of time, lack of interest, lack of motivation, and all other factors such as not feeling comfortable to do something, or any other reason.**

**231. In the past 30 days, how much difficulty have you had compared to other men/women of your age doing the following…**

**232.Taking care of your personal appearance**

Choose one response

- No more

- A little bit more

- A moderate amount more

- A lot more

- So much more that I often cannot do

- N/A

- No response

**233.Taking care of your health**

Choose one response

- No more

- A little bit more

- A moderate amount more

- A lot more

- So much more that I often cannot do

- N/A

- No response

**234.Spending time with your family**

Choose one response

- No more

- A little bit more

- A moderate amount more

- A lot more

- So much more that I often cannot do

- N/A

- No response

**235.Becoming part of the community**

Choose one response

- No more

- A little bit more

- A moderate amount more

- A lot more

- So much more that I often cannot do

- N/A

- No response

**236.Taking care of children**

Choose one response

- No more

- A little bit more

- A moderate amount more

- A lot more

- So much more that I often cannot do

- N/A

- No response

**237.Earning money and looking for employment**

Choose one response

- No more

- A little bit more

- A moderate amount more

- A lot more

- So much more that I often cannot do

- N/A

- No response

**238.Helping your family**

Choose one response

- No more

- A little bit more

- A moderate amount more

- A lot more

- So much more that I often cannot do

- N/A

- No response

**239.Doing household activities (e.g. cooking, cleaning, washing)**

Choose one response

- No more

- A little bit more

- A moderate amount more

- A lot more

- So much more that I often cannot do

- N/A

- No response

**240.Joining in community activities and cultural events (e.g. going on walks, going to museums, going to the theater**

Choose one response

- No more

- A little bit more

- A moderate amount more

- A lot more

- So much more that I often cannot do

- N/A

- No response

**241.Learning to live in a new community**

Choose one response

- No more

- A little bit more

- A moderate amount more

- A lot more

- So much more that I often cannot do

- N/A

- No response

**242.Trying to live in given conditions**

Choose one response

- No more

- A little bit more

- A moderate amount more

- A lot more

- So much more that I often cannot do

- N/A

- No response

**243.Spending time with people like me**

Choose one response

- No more

- A little bit more

- A moderate amount more

- A lot more

- So much more that I often cannot do

- N/A

- No response

**244.Looking for services and benefits/Registration of benefits**

Choose one response

- No more

- A little bit more

- A moderate amount more

- A lot more

- So much more that I often cannot do

- N/A

- No response

**245.Receiving education/Attending trainings**

Choose one response

- No more

- A little bit more

- A moderate amount more

- A lot more

- So much more that I often cannot do

- N/A

- No response

**246.Helping others**

Choose one response

- No more

- A little bit more

- A moderate amount more

- A lot more

- So much more that I often cannot do

- N/A

- No response

**247.Doing sports/exercise**

Choose one response

- No more

- A little bit more

- A moderate amount more

- A lot more

- So much more that I often cannot do

- N/A

- No response

**248.Doing hobbies (e.g. making crafts, sewing, embroidery, hunting, fishing, reading, games)**

Choose one response

- No more

- A little bit more

- A moderate amount more

- A lot more

- So much more that I often cannot do

- N/A

- No response

**249.Conversing with others**

Choose one response

- No more

- A little bit more

- A moderate amount more

- A lot more

- So much more that I often cannot do

- N/A

- No response

**250.Spending time in nature (e.g. working on land, walking outdoors)**

Choose one response

- No more

- A little bit more

- A moderate amount more

- A lot more

- So much more that I often cannot do

- N/A

- No response

**251.Making/keeping friends**

Choose one response

- No more

- A little bit more

- A moderate amount more

- A lot more

- So much more that I often cannot do

- N/A

- No response

**252.Creating conditions for comfortable living**

Choose one response

- No more

- A little bit more

- A moderate amount more

- A lot more

- So much more that I often cannot do

- N/A

- No response

**253. Thank you for your responses to the above questions. Please now hand the tablet back to the interviewer.**

**254. For the interviewer to read to the participant:**

**Now we are going to switch the focus of the questions. We are planning a program to address mental health problems of veterans, IDPs, and families and we are looking for local input on what a good program would look like. We are doing this by providing an example of a possible program and your responses to this description will help us design a better program. For the following questions, please read the description of the mental health program and then answer the questions based on your opinions as if you were a person who needed mental health services. If you do not have an opinion or there is not enough information, please mark "do not know." Answer to the best of your availability based on the information provided in the story. Where you feel like you cannot answer a question based on the information provided in the story, please note it and let the interviewer know about the problem at the end of the interview.**

**255.What would make you want to participate in this type of mental health program?**

**256.What would make you stop participating in this type of program if you had already started?**

**257.Would you discuss with others (e.g. family, friends, coworkers, or any other people) about what the mental health program is?**

Choose one response

- Not at all

- A little bit

- A moderate amount

- A lot

- Don't know

- Not enough information in story

**258.Would you discuss with others (e.g. family, friends, coworkers, or any other people) what you need to do if you participate in the mental health program?**

Choose one response

- Not at all

- A little bit

- A moderate amount

- A lot

- Don't know

- Not enough information in story

**259.Would you use the skills you learned in the mental health program in the future?**

Choose one response

- Not at all

- A little bit

- A moderate amount

- A lot

- Don't know

- Not enough information in story

**260.Would you encourage others to seek the mental health program’s services?**

Choose one response

- Not at all

- A little bit

- A moderate amount

- A lot

- Don't know

- Not enough information in story

**261.Would you refer others with similar problems to the mental health program?**

Choose one response

- Not at all

- A little bit

- A moderate amount

- A lot

- Don't know

- Not enough information in story

**262.Would you to return to the mental health services if you felt like you needed them in the future?**

Choose one response

- Not at all

- A little bit

- A moderate amount

- A lot

- Don't know

- Not enough information in story

**263.Overall, do you like the mental health program described?**

Choose one response

- Not at all

- A little bit

- A moderate amount

- A lot

- Don't know

- Not enough information in story

**264.Would you like attending the mental health treatment sessions as part of the program?**

Choose one response

- Not at all

- A little bit

- A moderate amount

- A lot

- Don't know

- Not enough information in story

**265.Would you feel satisfied with the mental health treatment in the program described?**

Choose one response

- Not at all

- A little bit

- A moderate amount

- A lot

- Don't know

- Not enough information in story

**266.Would you enjoy learning the mental health program described?**

Choose one response

- Not at all

- A little bit

- A moderate amount

- A lot

- Don't know

- Not enough information in story

**267.Would you feel like the skills you learned in the mental health program are useful?**

Choose one response

- Not at all

- A little bit

- A moderate amount

- A lot

- Don't know

- Not enough information in story

**268.Do you feel like the components (i.e. the strategies you learn in session) of the mental health program make sense?**

Choose one response

- Not at all

- A little bit

- A moderate amount

- A lot

- Don't know

- Not enough information in story

**269.Would you feel comfortable raising questions to your counselor if you participated in the mental health program?**

Choose one response

- Not at all

- A little bit

- A moderate amount

- A lot

- Don't know

- Not enough information in story

**270.Would you feel that the counselor in the mental health program would listen to your concerns and questions about the program?**

Choose one response

- Not at all

- A little bit

- A moderate amount

- A lot

- Don't know

- Not enough information in story

**271.Would you feel satisfied with your counselor’s abilities in the mental health program?**

Choose one response

- Not at all

- A little bit

- A moderate amount

- A lot

- Don't know

- Not enough information in story

**272.Would you feel that your counselor would address any of your questions or concerns about the program?**

Choose one response

- Not at all

- A little bit

- A moderate amount

- A lot

- Don't know

- Not enough information in story

**273.Would you feel that your counselor was available when you needed to talk to him/her?**

Choose one response

- Not at all

- A little bit

- A moderate amount

- A lot

- Don't know

- Not enough information in story

**274.Would you feel that you could trust your counselor?**

Choose one response

- Not at all

- A little bit

- A moderate amount

- A lot

- Don't know

- Not enough information in story

**275.How well does the mental health program described fit with your cultural values?**

Choose one response

- Not at all

- A little bit

- A moderate amount

- A lot

- Don't know

- Not enough information in story

**276.How well does the mental health program described fits with your personal values?**

Choose one response

- Not at all

- A little bit

- A moderate amount

- A lot

- Don't know

- Not enough information in story

**277.How well would participating in the mental health program fit into your daily schedule and routine?**

Choose one response

- Not at all

- A little bit

- A moderate amount

- A lot

- Don't know

- Not enough information in story

**278.How well does the mental health program described fit with the male culture in your country?**

Choose one response

- Not at all

- A little bit

- A moderate amount

- A lot

- Don't know

- Not enough information in story

**279.How well does the mental health program described fit with the female culture in your country?**

Choose one response

- Not at all

- A little bit

- A moderate amount

- A lot

- Don't know

- Not enough information in story

**280.Do you feel that the organization(s) providing the mental health program is a good place for delivery of these services?**

Choose one response

- Not at all

- A little bit

- A moderate amount

- A lot

- Don't know

- Not enough information in story

**281.Would you feel comfortable with the location where you would meet a counselor for this program?**

Choose one response

- Not at all

- A little bit

- A moderate amount

- A lot

- Don't know

- Not enough information in story

**282.Do you think the mental health program would be a good way to address your problems?**

Choose one response

- Not at all

- A little bit

- A moderate amount

- A lot

- Don't know

- Not enough information in story

**283.Do you think you would learn helpful strategies from the program to deal with your problematic thoughts, feelings and behaviors?**

Choose one response

- Not at all

- A little bit

- A moderate amount

- A lot

- Don't know

- Not enough information in story

**284.Do you think the mental health program would help you with your problems?**

Choose one response

- Not at all

- A little bit

- A moderate amount

- A lot

- Don't know

- Not enough information in story

**285.Do you think the mental health program would be appropriate for helping other people with similar problems as yours?**

Choose one response

- Not at all

- A little bit

- A moderate amount

- A lot

- Don't know

- Not enough information in story

**286.Do you think the mental health program addresses problems that are common in your community?**

Choose one response

- Not at all

- A little bit

- A moderate amount

- A lot

- Don't know

- Not enough information in story

**287.Do you think the skills taught in the mental health program would be relevant to other people like yourself?**

Choose one response

- Not at all

- A little bit

- A moderate amount

- A lot

- Don't know

- Not enough information in story

**288.Do you think you would have the necessary time to attend 8-12 weekly sessions of the mental health program?**

Choose one response

- Not at all

- A little bit

- A moderate amount

- A lot

- Don't know

- Not enough information in story

**289.Do you think the sessions of the mental health program would be scheduled with enough flexibility to meet your needs?**

Choose one response

- Not at all

- A little bit

- A moderate amount

- A lot

- Don't know

- Not enough information in story

**290.Do you think the counselor would be on time when you would come to sessions?**

Choose one response

- Not at all

- A little bit

- A moderate amount

- A lot

- Don't know

- Not enough information in story

**291.How possible would it be to get away from your duties (e.g. work, parenting) to attend the mental health program?**

Choose one response

- Not at all

- A little bit

- A moderate amount

- A lot

- Don't know

- Not enough information in story

**292.Do you think the amount of time you would spend each week on homework for the mental health program would be manageable?**

Choose one response

- Not at all

- A little bit

- A moderate amount

- A lot

- Don't know

- Not enough information in story

**293.Do you think you would have enough money to pay for transport to regularly attend the mental health program?**

Choose one response

- Not at all

- A little bit

- A moderate amount

- A lot

- Don't know

- Not enough information in story

**294.Do you think you would have enough money to pay for the other things you would need to regularly attend the mental health program?**

Choose one response

- Not at all

- A little bit

- A moderate amount

- A lot

- Don't know

- Not enough information in story

**295.How much would receiving the mental health service described affect your income?**

Choose one response

- Not at all

- A little bit

- A moderate amount

- A lot

- Don't know

- Not enough information in story

**296.Do you think you would have the resources (phone, talk time) to communicate with your counselor from the mental health program when needed?**

Choose one response

- Not at all

- A little bit

- A moderate amount

- A lot

- Don't know

- Not enough information in story

**297.Do you think you would have the emotional support you needed from your family and friends to regularly attend the mental health program?**

Choose one response

- Not at all

- A little bit

- A moderate amount

- A lot

- Don't know

- Not enough information in story

**298.How much would childcare responsibilities affect your ability to attend the mental health program?**

Choose one response

- Not at all

- A little bit

- A moderate amount

- A lot

- Don't know

- Not enough information in story

**299.In general, how safe would you feel to travel to weekly sessions for the mental health program?**

Choose one response

- Not at all

- A little bit

- A moderate amount

- A lot

- Don't know

- Not enough information in story

**300.How safe is the place where you would meet your counselor?**

Choose one response

- Not at all

- A little bit

- A moderate amount

- A lot

- Don't know

- Not enough information in story

**301.Do you think the place where you would meet your counselor would be confidential?**

Choose one response

- Not at all

- A little bit

- A moderate amount

- A lot

- Don't know

- Not enough information in story

**302.Do you believe people in your community could seek help for mental health problems from the program without fear of how others would view them?**

Choose one response

- Not at all

- A little bit

- A moderate amount

- A lot

- Don't know

- Not enough information in story

**303.Would people in the community be aware that the mental health program is available?**

Choose one response

- Not at all

- A little bit

- A moderate amount

- A lot

- Don't know

- Not enough information in story

**304.If you sought help, how much of a problem would you have with the amount of time you had to wait to begin the program?**

Choose one response

- Not at all

- A little bit

- A moderate amount

- A lot

- Don't know

- Not enough information in story

**305.Would most people in the community who need mental health services seek out the services provided by the program?**

Choose one response

- Not at all

- A little bit

- A moderate amount

- A lot

- Don't know

- Not enough information in story

**306.Would the poorest people in the community who need mental heath services seek out the services provided by the program?**

Choose one response

- Not at all

- A little bit

- A moderate amount

- A lot

- Don't know

- Not enough information in story

**307.What are reasons the poorest people would not seek services?**

**308.Would women who need mental health services seek out the services provided by the program?**

Choose one response

- Not at all

- A little bit

- A moderate amount

- A lot

- Don't know

- Not enough information in story

**309.What are reasons women would not seek services?**

**310.Would men who need mental heath services seek out the services provided by the program?**

Choose one response

- Not at all

- A little bit

- A moderate amount

- A lot

- Don't know

- Not enough information in story

**311.What are reasons men would not seek services?**

**312.Would parents or other caretakers seek the mental health services provided by the program if their children needed it?**

Choose one response

- Not at all

- A little bit

- A moderate amount

- A lot

- Don't know

- Not enough information in story

**313.What are reasons parents or caretakers would not seek services?**

**314.Would children without parents seek out the services provided by the program?**

Choose one response

- Not at all

- A little bit

- A moderate amount

- A lot

- Don't know

- Not enough information in story

**315.What are reasons children without parents would not seek services?**

**316.Who in your community would not seek the mental health services provided by the program?**

**317. This is the end of the survey. Thank you for your time and responses. Please hand the materials, including the tablet, back to the interviewer.**

**318. This is the end of the survey. Please scroll forward to view any alert messages and upload the data.**

**319.Did the respondent indicate suicide ideation?**

Choose one response

- no

- yes

**320.For the interviewer:**

**Please select "yes" below to indicate that the survey is complete.**

Choose one response

- Yes

- No
